# A new grey quadratic polynomial model and its application in the COVID-19 in China

**DOI:** 10.1038/s41598-021-91970-1

**Published:** 2021-06-15

**Authors:** Jianbo Zhang, Zeyou Jiang

**Affiliations:** 1 Sichuan Academy of Medical Sciences and Sichuan Provincial People’s Hospital Central Laboratory, No. 32 West Section 2, First Ring Road, Chengdu, 610072 China; 2 Department of Clinical Laboratory, Affiliated Hospital of Chengdu University of Traditional Chinese Medicine, No. 39, 12 Bridge Road, Chengdu, 610072 China

**Keywords:** Diseases, Engineering

## Abstract

This paper develops a new grey prediction model with quadratic polynomial term. Analytical expressions of the time response function and the restored values of the new model are derived by using grey model technique and mathematical tools. With observations of the confirmed cases, the death cases and the recovered cases from COVID-19 in China at the early stage, the proposed forecasting model is developed. The computational results demonstrate that the new model has higher precision than the other existing prediction models, which show the grey model has high accuracy in the forecasting of COVID-19.

## Introduction

At the beginning of 2020, a new strain of coronavirus (COVID-19) was found from some patients in January 2020. This disease can lead to severe fever, and mainly acute respiratory failure syndrome^1^. It is proven that this coronavirus can be transmitted from person to person. The number of confirmed cases rose sharply since the January 2020, and governments have to promulgate various laws and policies to alleviate the spread of COVID-19. At now, the total confirmed cases has reached 137,866,311 cases all over the world. Moreover, there is no indication that the virus will disappear within a few months. Thus accurately prediction the tendency, particularly at the early stage of the disease, can give a guidance for the control and prevention of the coronavirus.

It is generally known that the statistical models like autoregressive model, moving average and autoregressive integrated moving average, and the computational intelligence methods are widely applied in COVID-19 diseases. Castillo and Melin^2^ described a hybrid intelligent approach for efficient and accurate prediction COVID-19 time series combining fuzzy logic and fractal theory. Publicly available datasets of 10 countries are used to establish the fuzzy model, and the results show the new model can be considered good studying the complexity of this epidemic diseases. Chimmula and Zhang^3^ proposed a new state-of-the-art Deep Learning forecasting model for COVID-19 outbreak in Canada. The possible trends and stopping time of COVID-19 in Canada are evaluated, and then compared transmission rates of Canada with Italy and USA. Anastassopoulou et al.^4^ used a Susceptible-Infectious-Recovered-Dead (SIDR) model to study the basic reproduction number, the per day infection mortality and the recovery rates of Hubei in China. Petropoulos and Makridakis^5^ introduced an objective method to predict the spread of confirmed cases, the number of deaths and recoveries of the COVID-19 under the assumption that the original data is reliable and the process of the disease following the past pattern. Shastri et al.^6^ used neural network with Stacked LSTM, Convolutional LSTM and Bi-directional LSTM to study the confirmed cases and the death cases of COVID-19 in USA and India. Wang et al.^7^ developed a deep learning method with rolling mechanism to forecast the epidemic trend for Russia, Peru and Iran. Hawas^8^ introduced the recurrent neural networks for forecasting the virus’s daily infection in Brazil with limited raw data. Yonar et al.^9^ estimated the number of COVID-19 epidemic cases of Turkey, Germany, United Kingdom, France, Italy, Russia, Canada and Japan by Box-Jenkins (ARIMA), curve estimation models and Brown/Holt linear exponential smoothing methods. Melin et al.^10^ presented a multiple ensemble neural network with fuzzy logic method for the COVID-19 cases in Mexico where the errors are significantly lower than traditional neural networks. Sun and Wang^11^ examined the data from January 23 to March 25 by ordinary differential equation model, which demonstrate that strongly controlled measured can minimize total infections. Castillo and Melin^12^ proposed a hybrid intelligent fuzzy fractal method for COVID-19 classification of countries. Additionally, Luo et al.^13^, Sahin and Sahin^14^, Zhao et al.^15^ used grey models to study the number of patients infected with COVID-19. The *Chaos, Solitons and Fractals* launched an open focus issue for understanding and mitigating the effects of the current pandemic^16^. For more details about this topic, the interested readers can refer to^17–23^. Moreover, the details of these work are summarized in Table 1.

**Table 1:** Studies on COVID-19 analysis and forecasting.

Ref	Description	Forecasting method	focus	Data amount
^2^	Computational intelligence method	A hybrid intelligent approach based on fractal theory and fuzzy logic	Belgium, China, France, Germany, Iran Italy, Spain, Turkey, UK, US	01/22/2020–03/31/2020
^10^	Computational intelligence method	Multiple ensemble neural network model with fuzzy response aggregation	Mexico	to 05/18/2020
^12^	Computational intelligence method	Hybrid intelligent fuzzy fractal approach	Austria, Bolivia, Brazil, Ecuador, Finland, Greece, India, Morocco, New Zealand, Norway, Poland, Russia, Singapore, Sweden, Switzerland	04/01/2020–07/12/2020
^21^	Computational intelligence method	RNN based Long Short Term Memory (LSTM) model	China, Australia, USA, others	01/12/2020–05/11/2020
^6^	Deep learning method	RNN based variants of long short term memory (LSTM)	India, USA	02/07/2020–07/07/2020
^7^	Deep learning method	Recurrent neural networks (RNNs)	Russia, Peru, Iran	02/22/2020–07/07/2020
^3^	State-of-the-art Deep Learning models	the Long short-term memory networks	Canada	20/22/2020–03/31/2020
^4^	Nonlinear dynamics system	Susceptible Infectious-Recovered-Dead (SIDR) model	China	01/11/2020–02/10/2020
^11^	Nonlinear dynamics system	Ordinary differential equation model	China	01/23/2020–03/25/2020
^18^	Nonlinear dynamics system	SEIDIUQHRD deterministic compartmental model	Russia, Brazil, India, Bangladesh	02/01/2020–05/08/2020
^23^	Nonlinear dynamics system	Susceptible-Exposed-Infected-Recovered (SEIR) model	China, Italy	01/22/2020–03/30/2020
^9^	Parameter model	Box-Jenkins (ARIMA) and Brown/Holt linear exponential smoothing methods	Turkey, Germany, UK, France, Italy, Russia, Canada, Japan	01/22/2020–03/22/2020
^17^	Parameter model	Autoregressive Integrated Moving Average (ARIMA) model	USA, UK, Italy, Spain, France, China, India	07/12/2020–09/11/2020
^22^	Parameter model	Exponential decay model (EDM)	China	02/27/2020–04/07/2020
^5^	Statistical method	Null	South Korea, Iran, and Europe	01/22/2020–03/11/2020
^13^	Grey prediction model	GRM(1,1) model	China, Italy, United Kingdom, Russian	01/23–02/06 03/10–03/21 04/11–04/25 06/01–08/12
^14^	Grey prediction model	Fractional nonlinear grey Bernoulli model	Italy, UK, USA	04/22/2020–05/22/2020
^15^	Grey Prediction model	Rolling grey Verhulst model	China	01/21/2020–02/20/2020

It can be seen that the neural network models and statistical prediction models are widely used to study the COVID-19, and the grey prediction model is relatively few. As we know, the statistical models often require a large amount of historical data, at least thirty or more datasets, which obey a certain distribution. The neural network method needs a substantial amount of datasets for training to obtain system optimized parameters. However, the transmission mechanism of COVID-19 is not very clear, especially in the early stage owing to the limited information available. Thus it is very important to select a favorable technique for prediction the trend of the COVID-19 with limited information. The grey prediction method, proposed by Deng Julong^24^, is an efficient and accuracy method for solving uncertain problems with limited information. In the classical grey model GM(1,1), the grey action quantity is a constant number, which is essentially a homogenous exponent model. When the raw of data is not a homogeneous exponent sequence, the model accuracy maybe low. So Cui et al.^25^, Xie et al.^26^ put forward a non-homogeneous grey model with grey action quantity is *bt*. Chen and Yu^27^ based on the work of^25,26^ proposed a non-homogenous grey prediction model termed as NGM(1,1,k,c) in their work where the grey action quantity is *bt* + *c*. The whitening equation, the time response function and the restored values of the model are all derived with the grey techniques and mathematical tools. This model can simulate a homogeneous exponential sequence, a non-homogeneous exponential sequence and a linear sequence. However, we discover this non-homogeneous grey prediction model sometimes has large error with some sequences. To further improve the effectiveness and applicableness of grey models, we generalized the non-homogeneous grey forecasting model to a grey prediction model with quadratic polynomial term in this work.

At the early stage, the spreading mechanism of the COVID-19 is not clear, and there is limited available data to collect for us. Thus it is important for us to select an appropriate method to deal with the COVID-19, and obtain acceptable results. Under this situation, the grey forecasting model is chose to study the confirmed cases, the death cases and the recovered cases of COVID-19 in China at the early stage. With the grey theory and mathematical analysis, the grey quadratic polynomial model GMQP(1,1) is systematically studied. The grey basic form, the system parameters, the time response function and the restored values are all derived. Based on these expressions, some special cases are all considered. Further, the new model is applied to study the confirmed cases, the death cases and the recovered cases from COVID-19 in China at the early stage. The computational results are compared with the classical grey model GM(1,1)^24^, the discrete grey model DGM(1,1)^28,29^, the non-homogeneous grey model NGM(1,1,k,c)^27,30^, the grey Verhulst model GVM(1,1)^31–34^ and the polynomial regression PR(2) in the application section. It is found that the new model outperforms the other prediction models and can obtain competitive results. In summary, the main contributions and originalities of this work are provided here. (1**)** The grey forecasting model with quadratic polynomial term is develop, which can solve quasi homogeneous and non-homogeneous exponential series, or even some fluctuating series. (2**)** The analytical solution of time response function and the matrix expression of system parameters are also determined by grey technique. (3**)** The proposed newly model is a general grey forecasting model, and the GM(1,1) model, the NGM(1,1,k) model and the NGM(1,1,k,c) model are all special cases of the proposed model. Moreover, the feasibility of the new model is verified through two examples. (4**)** The new model is used to study the confirmed cases, the death cases and the recovered cases of COVID-19 in China at the early stage, and results illustrate that the new model has higher precision than other forecasting models.

The rest of this paper is arranged as follows. Section 2 discusses the existing grey forecasting models. The details of the grey prediction model with quadratic polynomial term is given in Sect. 3. Section 4 provides some numerical examples. Applications are studied in the Sect. 5. Conclusions are placed in the last section.

## Some existing grey forecasting models

This section provides a brief overview of some grey forecasting models which will used in the application section. They are the classical grey model GM(1,1), the discrete grey model DGM(1,1), the non-homogeneous grey model NGM(1,1,*k*,*c*) and the grey Verhulst model GVM(1,1). For concise, we only provide the whitening equation, the time response function and the restored values of them.

1. The GM(1,1) model
  The classical grey model GM(1,1) is the core of the grey forecasting theory. Since been putted forward, it has been widely applied in various fields including energy, economy and education. The whitening equation of GM(1,1) model is given by
  1 $$\frac{{dx^{\left( 1 \right)} \left( t \right)}}{dt} + ax^{\left( 1 \right)} \left( t \right) = b$$
  The time response function and the restored values are
  2 $$\hat{x}^{\left( 1 \right)} \left( k \right) = e^{{ - a\left( {k - 1} \right)}} \left( {x^{\left( 1 \right)} \left( 0 \right) - \frac{b}{a}} \right) + \frac{b}{a}$$
  3 $$\hat{x}^{\left( 0 \right)} \left( k \right) = e^{{ - a\left( {k - 2} \right)}} \left( {x^{\left( 1 \right)} \left( 0 \right) - \frac{b}{a}} \right)\left( {e^{a} - 1} \right)$$
2. The DGM(1,1) model
  The discrete grey forecasting model DGM(1,1) is initially provided by Xie and Liu^28,29^, the mathematical expression is
  4 $$x^{\left( 1 \right)} \left( k \right) = ax^{\left( 1 \right)} \left( {k - 1} \right) + b$$
  and the recursive function is given by
  5 $$\hat{x}^{\left( 1 \right)} \left( k \right) = a^{k - 1} x^{\left( 0 \right)} \left( 1 \right) + \frac{{1 - a^{k - 1} }}{1 - a}b$$
3. The NGM(1,1,k,c) model
  The whitening equation of the NGM(1,1,k,c) is
  6 $$\frac{{dx^{\left( 1 \right)} \left( t \right)}}{dt} + ax^{\left( 1 \right)} \left( t \right) = bt + c$$
  The time response function and the restored values are
  7 $$\hat{x}^{\left( 1 \right)} \left( k \right) = e^{{ - a\left( {k - 1} \right)}} \left( {x^{\left( 1 \right)} \left( 0 \right) - \frac{b}{a} + \frac{b}{{a^{2} }} - \frac{c}{a}} \right) + \frac{b}{a}k - \frac{b}{{a^{2} }} + \frac{c}{a}$$
  8 $$\hat{x}^{\left( 0 \right)} \left( k \right) = e^{{ - a\left( {k - 2} \right)}} \left( {x^{\left( 1 \right)} \left( 0 \right) - \frac{b}{a} + \frac{b}{{a^{2} }} - \frac{c}{a}} \right)\left( {e^{a} - 1} \right) + \frac{b}{a}$$
4. The GVM(1,1) model.
  This nonlinear grey model is first appeared in the book of Deng^34^, which is able to simulate and predict original observations with an inverted U shape or a signal peak feature. The whitening equation of GVM(1,1) model is
  9 $$\frac{{dx^{\left( 1 \right)} \left( t \right)}}{dt} + ax^{\left( 1 \right)} \left( t \right) = b\left( {x^{\left( 1 \right)} \left( t \right)} \right)^{2}$$
  Further, the time response function and the restored values are
  10 $$\hat{x}^{\left( 1 \right)} \left( k \right) = \frac{1}{{\frac{b}{a} + \left( {\frac{1}{{x^{\left( 0 \right)} \left( 1 \right)}} - \frac{b}{a}} \right)e^{{a\left( {k - 1} \right)}} }}$$
  11 $$\hat{x}^{\left( 0 \right)} \left( k \right) = \left\{ {\begin{array}{*{20}c} {x^{\left( 0 \right)} \left( 1 \right),\quad \quad \quad \quad } \\ {\hat{x}^{\left( 1 \right)} \left( k \right) - \hat{x}^{\left( 1 \right)} \left( {k - 1} \right)} \\ \end{array} } \right.$$

## The grey model with quadratic polynomial term

This section discusses the grey model with quadratic polynomial term which is abbreviated as GMQP(1,1) model in the present paper. We first provide the definition of the accumulated and inverse accumulated generation operators, and then discuss the new model GMQP(1,1) along with some properties.

### Accumulated and inverse accumulated generation operator

#### Definition 1

(Accumulated generation operator) First, we assume the original non-negative sequence is $X^{\left( 0 \right)} = \left( {x^{\left( 0 \right)} \left( 1 \right),x^{\left( 0 \right)} \left( 2 \right), \cdots ,x^{\left( 0 \right)} \left( n \right)} \right)$, and A is a sequence operator such that $X^{\left( 0 \right)} A = X^{\left( 1 \right)} = \left( {x^{\left( 1 \right)} \left( 1 \right),x^{\left( 1 \right)} \left( 2 \right), \cdots ,x^{\left( 1 \right)} \left( n \right)} \right)$, where the relationship is given by $x^{\left( 1 \right)} \left( k \right) = \sum\limits_{i = 1}^{k} {x^{\left( 0 \right)} \left( i \right)} ,k = 1,2, \cdots ,n$. The operator A is named as the first-order accumulated generation operator (1-AGO) of original sequence $X^{\left( 0 \right)}$.

It follows from definition 1 that $X^{\left( m \right)} = X^{\left( 0 \right)} A^{m} = \left( {x^{\left( m \right)} \left( 1 \right),x^{\left( m \right)} \left( 2 \right), \cdots ,x^{\left( m \right)} \left( n \right)} \right)$, $m = 1,2, \cdots$ where $x^{\left( m \right)} \left( k \right) = \sum\limits_{i = 1}^{k} {x^{{\left( {m - 1} \right)}} \left( i \right)} ,k = 1,2, \cdots ,n$.

#### Definition 2

(Inverse accumulated generation operator). The inverse accumulated generation operator is defined as $X^{{\left( { - m} \right)}} = X^{\left( 0 \right)} D^{m} = \left( {x^{{\left( { - m} \right)}} \left( 1 \right),x^{{\left( { - m} \right)}} \left( 2 \right), \cdots ,x^{{\left( { - m} \right)}} \left( n \right)} \right),m = 1,2, \cdots$, where $x^{{\left( { - m} \right)}} \left( k \right) = x^{{\left( {m - 1} \right)}} \left( k \right) - x^{{\left( {m - 1} \right)}} \left( {k - 1} \right),k = 2, \cdots ,n$ and $x^{{\left( { - m} \right)}} \left( 1 \right) = x^{\left( 0 \right)} \left( 1 \right)$.

It follows from the definition 1 and definition 2 that the inverse accumulated generation operator is the inverse operation of the accumulated generation operator.

### The grey quadratic polynomial model

#### Definition 3

Assume $X^{\left( 0 \right)}$ and $X^{\left( 1 \right)}$ are stated in definition 1, then the whitening differential equation of the grey model with quadratic polynomial term is defined as.

12 $$\frac{{dx^{\left( 1 \right)} \left( t \right)}}{dt} + ax^{\left( 1 \right)} \left( t \right) = bt^{2} + ct + d$$

where *a* is the development coefficient, and $bt^{2} + ct + d$ is the grey action quantity.

Obviously, when system parameter *b* = 0 in Eq. (12), the GMQP(1,1) model degenerates to the NGM(1,1,*k*,*c*) model.

When the parameters *b* = 0 and *c* = 0 in Eq. (12), the GMQP(1,1) model reduces to the classical GM(1,1) model.

#### Theorem 1

The basic form of the GMQP(1,1) model is represented by.

13 $$x^{\left( 0 \right)} \left( k \right) + az^{\left( 1 \right)} \left( k \right) = \left( {k^{2} - k + 1/3} \right)b + \left( {k - 1/2} \right)c + d$$

where $z^{\left( 1 \right)} \left( k \right) = 0.5 \times \left( {x^{\left( 1 \right)} \left( {k - 1} \right) + x^{\left( 1 \right)} \left( k \right)} \right),k = 2,3, \cdots ,n$ is called the mean sequence or background values.

#### Proof

The whitening equation is integral on interval [*k*-1, *k*],

14 $$\int_{k - 1}^{k} {dx^{\left( 1 \right)} \left( t \right)} + \int_{k - 1}^{k} {ax^{\left( 1 \right)} \left( t \right)dt} = \int_{k - 1}^{k} {bt^{2} dt} + \int_{k - 1}^{k} {ctdt} + \int_{k - 1}^{k} {ddt}$$

It yields that

15 $$x^{\left( 0 \right)} \left( k \right) + a\int_{k - 1}^{k} {x^{\left( 1 \right)} \left( t \right)dt} = b\frac{{k^{3} - \left( {k - 1} \right)^{3} }}{3} + c\frac{{k^{2} - \left( {k - 1} \right)^{2} }}{2} + d$$

With the trapezoid formula $\int_{k - 1}^{k} {x^{\left( 1 \right)} \left( t \right)dt} = \frac{{x^{\left( 1 \right)} \left( {k - 1} \right) + x^{\left( 1 \right)} \left( k \right)}}{2} = z^{\left( 1 \right)} \left( k \right)$, and some mathematical calculations, we have

16 $$x^{\left( 0 \right)} \left( k \right) + az^{\left( 1 \right)} \left( k \right) = \left( {k^{2} - k + 1/3} \right)b + \left( {k - 1/2} \right)c + d$$

this completes the proof.

#### Theorem 2

Let raw data sequence $X^{\left( 0 \right)} = \left( {x^{\left( 0 \right)} \left( 1 \right),x^{\left( 0 \right)} \left( 2 \right), \cdots ,x^{\left( 0 \right)} \left( n \right)} \right)$ be the non-negative sequence, $X^{\left( 1 \right)} = \left( {x^{\left( 1 \right)} \left( 1 \right),x^{\left( 1 \right)} \left( 2 \right), \cdots ,x^{\left( 1 \right)} \left( n \right)} \right)$ is the 1-AGO sequence of $X^{\left( 0 \right)}$, and the background value is $z^{\left( 1 \right)} \left( k \right)$. The column parameter $\left( {a,b,c,d} \right)^{T}$ of the GMQP(1,1) model is presented by the following relationship.

17 $$\left( {a,b,c,d} \right)^{T} = \left( {B^{T} B} \right)^{ - 1} B^{T} Y$$

where

$$B = \left( {\begin{array}{*{20}c} { - z^{\left( 1 \right)} \left( 2 \right)} & \frac{7}{3} & \frac{3}{2} & 1 \\ { - z^{\left( 1 \right)} \left( 3 \right)} & \frac{19}{3} & \frac{5}{2} & 1 \\ \vdots & \vdots & \vdots & \vdots \\ { - z^{\left( 1 \right)} \left( n \right)} & {n^{2} - n + \frac{1}{3}} & {n - \frac{1}{2}} & 1 \\ \end{array} } \right),Y = \left( {\begin{array}{*{20}c} {x^{\left( 0 \right)} \left( 2 \right)} \\ {x^{\left( 0 \right)} \left( 3 \right)} \\ \vdots \\ {x^{\left( 0 \right)} \left( n \right)} \\ \end{array} } \right)$$

#### Proof

Employing the mathematical induction considering *k* = 2,3,…,*n* into Theorem 1, we obtain that.

$$\left\{ {\begin{array}{*{20}c} { - az^{\left( 1 \right)} \left( 2 \right) + \frac{7}{3}b + \frac{3}{2}c + d = x^{\left( 0 \right)} \left( 2 \right),\quad \quad \quad \quad \quad \quad } \\ { - az^{\left( 1 \right)} \left( 3 \right) + \frac{19}{3}b + \frac{5}{2}c + d = x^{\left( 0 \right)} \left( 3 \right),\quad \quad \quad \quad \quad \quad } \\ \vdots \\ { - az^{\left( 1 \right)} \left( n \right) + \left( {n^{2} - n + \frac{1}{3}} \right)b + \left( {n - \frac{1}{2}} \right)c + d = x^{\left( 0 \right)} \left( n \right)} \\ \end{array} } \right.$$.

Converting the above equation system into the matrix form, we can get

18 $$\left( {\begin{array}{*{20}c} { - z^{\left( 1 \right)} \left( 2 \right)} & \frac{7}{3} & \frac{3}{2} & 1 \\ { - z^{\left( 1 \right)} \left( 3 \right)} & \frac{19}{3} & \frac{5}{2} & 1 \\ \vdots & \vdots & \vdots & \vdots \\ { - z^{\left( 1 \right)} \left( n \right)} & {n^{2} - n + \frac{1}{3}} & {n - \frac{1}{2}} & 1 \\ \end{array} } \right)\left( {\begin{array}{*{20}c} a \\ b \\ c \\ d \\ \end{array} } \right) = \left( {\begin{array}{*{20}c} {x^{\left( 0 \right)} \left( 2 \right)} \\ {x^{\left( 0 \right)} \left( 3 \right)} \\ \vdots \\ {x^{\left( 0 \right)} \left( n \right)} \\ \end{array} } \right)$$

It is easily known that $\left( {a,b,c,d} \right)^{T} = \left( {B^{T} B} \right)^{ - 1} B^{T} Y$.

#### Theorem 3

The analytical expression of the time response sequence of the GMQP(1,1) model is given by.

19 $$\begin{gathered} \hat{x}^{\left( 1 \right)} \left( k \right) = e^{{ - a\left( {k - 1} \right)}} \left( {x^{\left( 1 \right)} \left( 0 \right) - \frac{b}{a} + \frac{2b}{{a^{2} }} - \frac{2b}{{a^{3} }} - \frac{c}{a} + \frac{c}{{a^{2} }} - \frac{d}{a}} \right) \hfill \\ \quad \quad \quad \quad + \frac{b}{a}k^{2} - \left( {\frac{2b}{{a^{2} }} - \frac{c}{a}} \right)k + \frac{2b}{{a^{3} }} - \frac{c}{{a^{2} }} + \frac{d}{a} \hfill \\ \end{gathered}$$

and the restored values $\hat{x}^{\left( 0 \right)} \left( k \right)$ can be derived by utilizing the 1-IAGO, that is

20 $$\begin{gathered} \hat{x}^{\left( 0 \right)} \left( k \right) = e^{{ - a\left( {k - 2} \right)}} \left( {x^{\left( 1 \right)} \left( 0 \right) - \frac{b}{a} + \frac{2b}{{a^{2} }} - \frac{2b}{{a^{3} }} - \frac{c}{a} + \frac{c}{{a^{2} }} - \frac{d}{a}} \right)\left( {e^{a} - 1} \right) \hfill \\ \quad \quad \quad \quad + \frac{2b}{a}k - \frac{b}{a} - \frac{2b}{{a^{2} }} + \frac{c}{a} \hfill \\ \end{gathered}$$

#### Proof

It follows from the theory of the ordinary differential equation that the general solution of the whitening equation is

21 $$\begin{gathered} x^{\left( 1 \right)} \left( t \right) = x^{\left( 1 \right)} \left( 0 \right)e^{{ - \int_{1}^{t} a d\tau }} + \int_{1}^{t} {\left( {bs^{2} + cs + d} \right)e^{{\int_{t}^{s} a d\tau }} } ds \hfill \\ \quad \quad \;\; = x^{\left( 1 \right)} \left( 0 \right)e^{{ - a\left( {t - 1} \right)}} + e^{ - at} \left( {b\int_{1}^{t} {s^{2} e^{as} } ds + c\int_{1}^{t} {se^{as} } ds + d\int_{1}^{t} {e^{as} } ds} \right) \hfill \\ \end{gathered}$$

Noting that $\int_{1}^{t} {e^{as} } ds = \frac{1}{a}\left( {e^{at} - e^{a} } \right)$,$\int_{1}^{t} {se^{as} } ds = e^{at} \left( {\frac{t}{a} - \frac{1}{{a^{2} }}} \right) - e^{a} \left( {\frac{1}{a} - \frac{1}{{a^{2} }}} \right)$ and $\int_{1}^{t} {s^{2} e^{as} } ds = e^{at} \left( {\frac{{t^{2} }}{a} - \frac{2t}{{a^{2} }} + \frac{2}{{a^{3} }}} \right) - e^{a} \left( {\frac{1}{a} - \frac{2}{{a^{2} }} + \frac{2}{{a^{3} }}} \right)$, we can obtain

22 $$\begin{gathered} x^{\left( 1 \right)} \left( t \right) = e^{{ - a\left( {t - 1} \right)}} \left( {x^{\left( 1 \right)} \left( 0 \right) - \frac{b}{a} + \frac{2b}{{a^{2} }} - \frac{2b}{{a^{3} }} - \frac{c}{a} + \frac{c}{{a^{2} }} - \frac{d}{a}} \right) \hfill \\ \quad \quad \;\; + \frac{b}{a}t^{2} - \left( {\frac{2b}{{a^{2} }} - \frac{c}{a}} \right)t + \frac{2b}{{a^{3} }} - \frac{c}{{a^{2} }} + \frac{d}{a} \hfill \\ \end{gathered}$$

Finally, we can discrete the expression of $x^{\left( 1 \right)} \left( t \right)$ to get the time response function, and the restored values $\hat{x}^{\left( 0 \right)} \left( k \right)$ of the GMQP(1,1) model.

### Error checking method

The performance of model should include two aspects: the simulation performance and the fitting performance.

Assume a raw sequence $X^{\left( 0 \right)} = \left( {x^{\left( 0 \right)} \left( 1 \right),x^{\left( 0 \right)} \left( 2 \right), \cdots ,x^{\left( 0 \right)} \left( m \right),x^{\left( 0 \right)} \left( {m + 1} \right), \ldots ,x^{\left( 0 \right)} \left( n \right)} \right)$ where a subsequence composed of the first *m* entries of raw sequence $X^{\left( 0 \right)}$ is applied to develop the newly proposed model, and simulation sequence is $\hat{X}_{S}^{\left( 0 \right)} = \left( {\hat{x}^{\left( 0 \right)} \left( 1 \right),\hat{x}^{\left( 0 \right)} \left( 2 \right), \cdots ,\hat{x}^{\left( 0 \right)} \left( m \right)} \right)$. We utilize the grey forecasting model to forecast the left *n*-*m* steps data, and the prediction sequence is $X_{F}^{\left( 0 \right)} = \left( {\hat{x}^{\left( 0 \right)} \left( {m + 1} \right),\hat{x}^{\left( 0 \right)} \left( {m + 2} \right), \cdots ,\hat{x}^{\left( 0 \right)} \left( n \right)} \right)$.

The error sequence of the simulation sequence $\hat{X}_{S}^{\left( 0 \right)}$ and the prediction sequence $\hat{X}_{F}^{\left( 0 \right)}$ are, respectively, $\varepsilon_{S}$ and $\varepsilon_{F}$, which are given as follows

$$\varepsilon_{S} = \left( {\varepsilon_{S} \left( 1 \right),\varepsilon_{S} \left( 2 \right), \cdots ,\varepsilon_{S} \left( m \right)} \right),\varepsilon_{F} = \left( {\varepsilon_{F} \left( {m + 1} \right),\varepsilon_{F} \left( {m + 2} \right), \cdots ,\varepsilon_{F} \left( n \right)} \right)$$

where $$\varepsilon_{S} \left( u \right) = \left| {x^{\left( 0 \right)} \left( u \right) - \hat{x}^{\left( 0 \right)} \left( u \right)} \right|,u = 1,2, \ldots ,m$$ and $$\varepsilon_{F} \left( u \right) = \left| {x^{\left( 0 \right)} \left( u \right) - \hat{x}^{\left( 0 \right)} \left( u \right)} \right|,u = m + 1,m + 2, \ldots ,n$$.

Here the absolute percentage error (APE), the absolute error (MAE), the mean squares error (MSE), the mean absolute percentage error (MAPE), the root mean square percentage error (RMSPE), the index of agreement (IA) and the correlation coefficient (R) are provided below.

**Figure 1:**
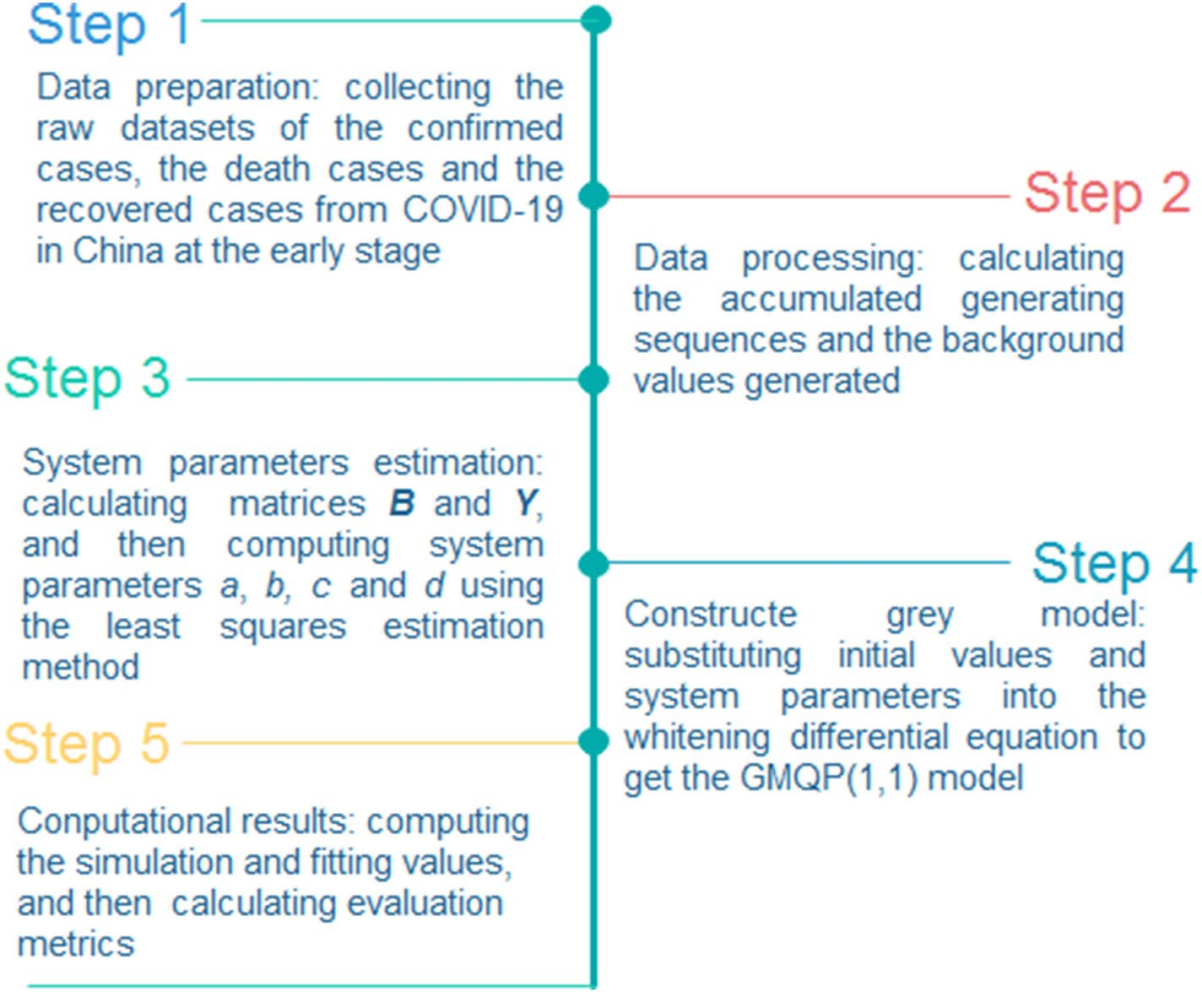
The flowchart of the GMQP(1,1) model.

- The absolute percentage error
  23 $$APE\left( k \right) = \frac{{\varepsilon_{S/F} \left( k \right)}}{{x^{\left( 0 \right)} \left( k \right)}} \times 100\% ,k = 1,2, \ldots ,n$$
- The absolute error (MAE)
  24 $$MAE = \frac{1}{r - l + 1}\sum\limits_{k = l}^{r} {\varepsilon_{S/F} \left( k \right)} \times 100\%$$
  where $l = 1,r = m$ is the mean absolute simulation percentage error MAE_sim_, $l = m + 1,r = n$ is the mean absolute fitting percentage error MAE_fit_, $l = 1,r = n$ is the total mean absolute percentage error MAE_all_.
- The mean squares error (MSE)
  25 $$MSE = \frac{1}{r - l + 1}\sum\limits_{k = l}^{r} {\left[ {\varepsilon_{S/F} \left( k \right)} \right]^{2} } \times 100\%$$
- The mean absolute percentage error
  26 $$MAPE = \frac{1}{r - l + 1}\sum\limits_{k = l}^{r} {\frac{{\varepsilon_{S/F} \left( k \right)}}{{x^{\left( 0 \right)} \left( k \right)}}} \times 100\%$$
- The root mean square percentage error
  27 $$RMSPE = \sqrt {\frac{1}{r - l + 1}\sum\limits_{k = l}^{r} {\left( {\frac{{\varepsilon_{S/F} \left( k \right)}}{x\left( k \right)}} \right)^{2} } } \times 100\%$$
- The index of agreement (IA)
  28 $$IA = 1 - \frac{{\sum\limits_{k = l}^{r} {\left[ {\varepsilon_{S/F} \left( k \right)} \right]^{2} } }}{{\sum\limits_{k = l}^{r} {\left[ {\left| {\hat{x}^{\left( 0 \right)} \left( k \right) - \overline{x}} \right| + \left| {x^{\left( 0 \right)} \left( k \right) - \overline{x}} \right|} \right]^{2} } }}$$
  where $\overline{x}$ is the mean value of original sequence.
- The correlation coefficient (R)
  29 $$R = \frac{{{\text{cov}} \left( {\hat{X}^{\left( 0 \right)} ,X^{\left( 0 \right)} } \right)}}{{\sqrt {{\text{var}} \left( {\hat{X}^{\left( 0 \right)} } \right)} \sqrt {{\text{var}} \left( {X^{\left( 0 \right)} } \right)} }}$$
  Moreover, the flowchart of the GMQP(1,1) model is listed in the following Fig. 1.

## Validation of the GMQP(1,1) model

To validation of the feasibility of the new model, this section gives two numerical example where datasets are collected from published papers.

### Example 1

In this example, data are all collected from Table 2 in Ref^35^. where the total energy consumption in China (unit: 10000tce). These data are used to build the GM(1,1) model, the DGM(1,1) model, the NGM(1,1,k,c) model, the GVM(1,1) model and the GMQP(1,1) model. The numerical results of these grey forecasting models are displayed in the following Tables 2, 3 and 4.

**Table 2:** The numerical results of the energy consumption of China (unit: 10,000 tce).

year	values	GM(1,1)	DGM(1,1)	NGM(1,1,k,c)	GVM(1,1)	GMQP(1,1)
1999	140,568.82	140,568.8200	140,568.8200	140,568.8200	140,568.8200	140,568.8200
2000	145,530.86	154,097.5428	154,173.2782	121,587.3934	41,622.9108	131,646.4473
2001	150,405.8	166,155.7594	166,243.3504	142,213.0901	53,370.7429	152,246.2752
2002	159,430.99	179,157.5379	179,258.3764	162,614.7168	68,053.9908	172,683.2660
2003	183,791.82	193,176.7127	193,292.3359	182,794.7080	86,162.8285	192,937.8565
2004	213,455.99	208,292.8956	208,425.0000	202,755.4712	108,114.8464	212,988.1330
2005	235,996.65	224,591.9280	224,742.3853	222,499.3882	134,140.9801	232,809.5492
2006	258,676.3	242,166.3685	242,337.2425	242,028.8146	164,126.8251	252,374.6097
2007	280,507.94	261,116.0186	261,309.5834	261,346.0807	197,422.6059	271,652.5157
2008	291,448.29	281,548.4891	281,767.2500	280,453.4912	232,662.9912	290,608.7684
2009	306,647.15	303,579.8116	303,826.5269	299,353.3259	267,672.3260	309,204.7243
2010	324,939.15	327,335.0970	327,612.8026	318,047.8398	299,553.1267	327,397.0971
2011	348,001.66	352,949.2464	353,261.2821	336,539.2636	325,035.1990	345,137.4001
2012	361,732.01	380,567.7169	380,917.7554	354,829.8034	341,075.7973	362,371.3216

**Table 3:** The APEs of these forecasting models in the energy consumption of China.

year	GM(1,1)	DGM(1,1)	NGM(1,1,k,c)	GVM(1,1)	GMQP(1,1)
1999	0.0000	0.0000	0.0000	0.0000	0.0000
2000	5.8865	5.9385	16.4525	71.3993	9.5405
2001	10.4716	10.5299	5.4471	64.5155	1.2237
2002	12.3731	12.4363	1.9969	57.3145	8.3122
2003	5.1063	5.1692	0.5425	53.1193	4.9763
2004	2.4188	2.3569	5.0130	49.3503	0.2192
2005	4.8326	4.7688	5.7193	43.1598	1.3505
2006	6.3825	6.3164	6.4356	36.5513	2.4361
2007	6.9131	6.8441	6.8311	29.6196	3.1569
2008	3.3968	3.3217	3.7725	20.1701	0.2881
2009	1.0003	0.9198	2.3786	12.7100	0.8340
2010	0.7374	0.8228	2.1208	7.8125	0.7564
2011	1.4217	1.5114	3.2938	6.5995	0.8231
2012	5.2071	5.3039	1.9081	5.7104	0.1767

**Table 4:** The evaluation measures of these models in the energy consumption of China.

	GM(1,1)	DGM(1,1)	NGM(1,1,k,c)	GVM(1,1)	GMQP(1,1)
MAE	11,157.2409	11,173.1710	10,759.1290	72,426.8799	5099.5298
MSE	161,263,808.4429	161,615,292.1515	153,318,142.5998	6,306,252,770.1604	46,603,353.8470
MAPE	5.0883	5.0954	4.7624	35.2332	2.6226
RMSPE	6.0981	6.1114	6.1359	41.6799	3.9827
IA	0.9922	0.9922	0.9927	0.8182	0.9978
R	0.9869	0.9869	0.9955	0.9479	0.9962

We can from Tables 2, 3, and 4 that the new model has better performance measures than other grey forecasting models in the energy consumption of China, which show that the new structure of GMQP(1,1) model can improve the precision of grey model.

### Example 2

In this example, the raw data of the electricity consumption of China are collected from Table 2 in Ref.^36^, where the twelve data are all applied to build different kinds of grey models. Similarly, the computational results and evaluation measures are listed in the following Tables 5, 6, and 7.

**Table 5:** The results of the electricity of China by different grey forecasting models.

year	values	GM(1,1)	DGM(1,1)	NGM(1,1,k,c)	GVM(1,1)	GMQP(1,1)
2002	1654	1654.0000	1654.0000	1654.0000	1654.0000	1654.0000
2003	1910.5	2113.2755	2114.9688	1785.3338	574.2617	1908.7598
2004	2203.3	2325.4896	2327.5232	2084.6507	764.2721	2212.6902
2005	2500.2	2559.0142	2561.4393	2392.2518	1009.7824	2524.3652
2006	2865.7	2815.9893	2818.8640	2708.3663	1321.4159	2844.1501
2007	3281.5	3098.7696	3102.1598	3033.2299	1707.6737	3172.4276
2008	3495.7	3409.9466	3413.9269	3367.0848	2171.4243	3509.5984
2009	3714.6	3752.3719	3757.0266	3710.1797	2705.0199	3856.0821
2010	4192.3	4129.1834	4134.6078	4062.7704	3284.8796	4212.3181
2011	4692.8	4543.8341	4550.1359	4425.1198	3867.7067	4578.7665
2012	4959.1	5000.1238	5007.4246	4797.4979	4391.7255	4955.9092
2013	5322.3	5502.2339	5510.6708	5180.1823	4786.0089	5344.2508

**Table 6:** The APEs of these grey models in the electricity consumption in China.

year	GM(1,1)	DGM(1,1)	NGM(1,1,k,c)	GVM(1,1)	GMQP(1,1)
2002	0.0000	0.0000	0.0000	0.0000	0.0000
2003	10.6137	10.7024	6.5515	69.9418	0.0911
2004	5.5458	5.6381	5.3851	65.3124	0.4262
2005	2.3524	2.4494	4.3176	59.6119	0.9665
2006	1.7347	1.6344	5.4902	53.8885	0.7520
2007	5.5685	5.4652	7.5657	47.9606	3.3239
2008	2.4531	2.3392	3.6792	37.8830	0.3976
2009	1.0169	1.1422	0.1190	27.1787	3.8088
2010	1.5055	1.3761	3.0897	21.6449	0.4775
2011	3.1744	3.0401	5.7041	17.5821	2.4300
2012	0.8272	0.9745	3.2587	11.4411	0.0643
2013	3.3808	3.5393	2.6702	10.0763	0.4124

**Table 7:** The evaluation measures of these models in the electricity consumption of China.

	GM(1,1)	DGM(1,1)	NGM(1,1,k,c)	GVM(1,1)	GMQP(1,1)
MAE	106.6169	107.0326	144.6666	1141.2572	43.6811
MSE	14,943.9048	15,030.2529	25,372.1541	1,438,735.6144	4285.7338
MAPE	3.4703	3.4819	4.3483	38.4110	1.1955
RMSPE	4.4155	4.4339	4.7800	43.7962	1.7506
IA	0.9969	0.9968	0.9946	0.7980	0.9991
R	0.9950	0.9950	0.9982	0.9444	0.9986

It is shown that the GM(1,1) model, the DGM(1,1) model, the NGM(1,1,k,c) model and the GMQP(1,1) model successfully catch the trend of the electricity consumption of China. Moreover, the new model has the best performance measures, while the GVM(1,1) model has the worst performance measures.

It follows from example 1 and example 2 that the new grey model has best performance measures, which shows the new grey models with a more flexible structure can be a good way of improving the accuracy of model.

## Applications in the COVID-19 of China

**Figure 2:**
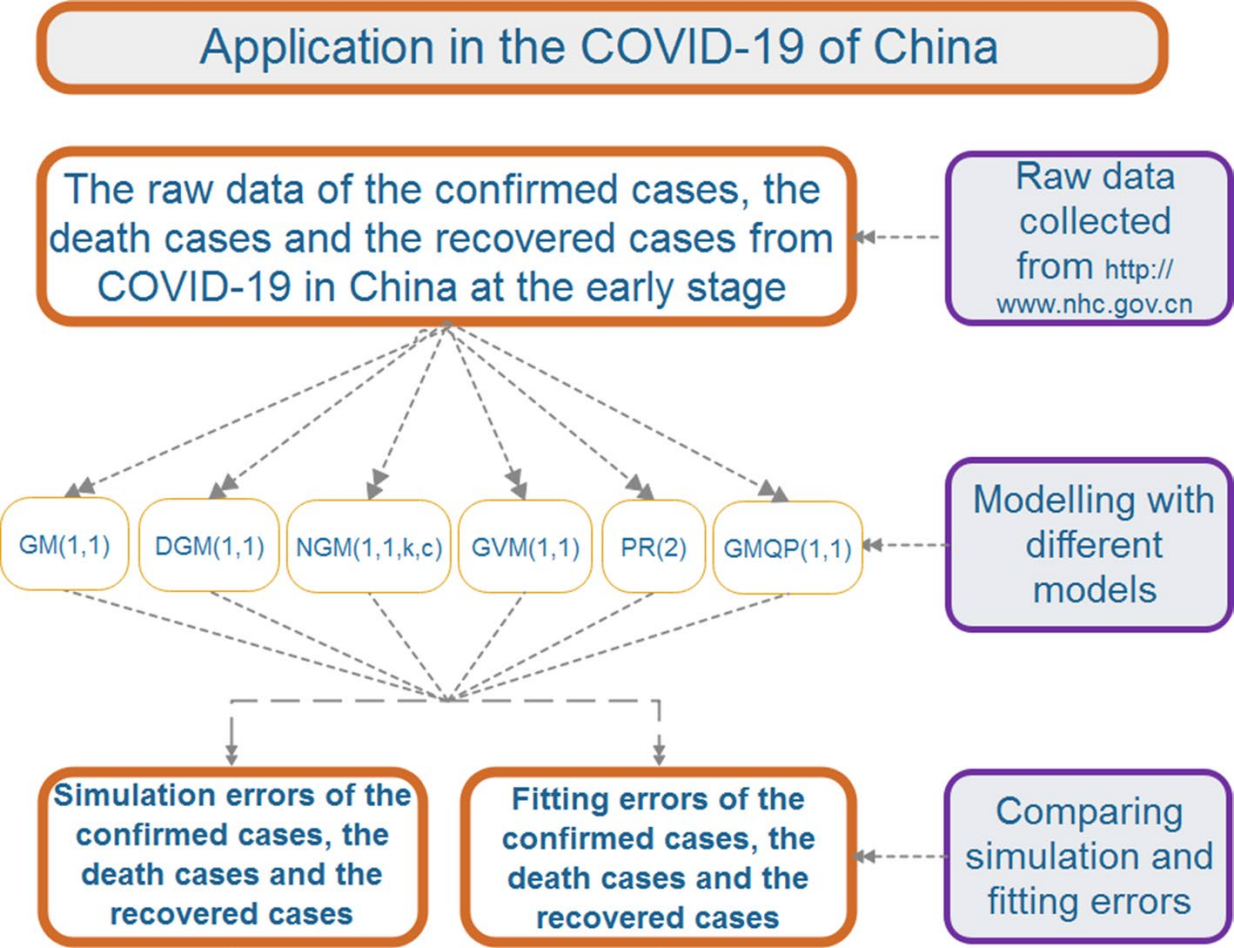
The structure of the application in the COVID-19 of China.

In this section, we will use different grey forecasting models and the polynomial regression to study the confirmed cases, the death cases and the recovered cases from COVID-19 in China, which are the classical continuous grey model GM(1,1), the discrete grey model DGM(1,1), the non-homogeneous grey model NGM(1,1,*k*,*c*), the nonlinear grey Verhulst model GVM(1,1), the polynomial regression (PR) and the grey model with quadratic polynomial term GMQP(1,1). Moreover, the structure of the applications in the COVID-19 of China is shown in Fig. 2.

### The confirmed cases from COVID-19 of China

**Figure 3:**
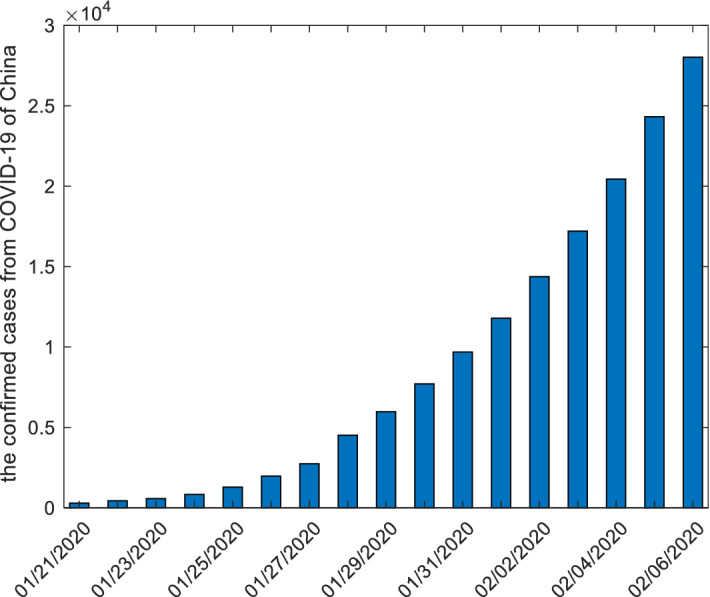
The plots of the confirmed cases from COVID-19 of China.

In this subsection, we apply forecasting models to study the confirmed cases from COVID-19 of China. The raw data, starting 2020-01-21 to 2020-02-06, are collected from the website: http://www.nhc.gov.cn, and displayed in the following Table 8 and Fig. 3.

**Table 8:** The number of the confirmed cases from COVID-19 of China.

date	01/21/2020	01/22/2020	01/23/2020	01/24/2020	01/25/2020	01/26/2020
raw data	291	440	571	830	1287	1975
date	01/27/2020	01/28/2020	01/29/2020	01/30/2020	01/31/2020	02/01/2020
raw data	2744	4515	5974	7711	9692	11,791
date	02/02/2020	02/03/2020	02/04/2020	02/05/2020	02/06/2020	
raw data	14,380	17,205	20,438	24,324	28,018	

With these raw data, we can deduce the mathematical expressions of different grey model. Here we take the GMQP(1,1) model as an example to details show the modelling procedures.

*Step 1* pre-process the raw data.

It follows from Table 8 that the original sequence is X^(0)^ = (291, 440, 571, 830, 1287, 1975, 2744, 4515, 5974, 7711, 9692, 11,791, 14,380, 17,205, 20,438, 24,324, 28,018). The first 14 data are used to develop the GMQP(1,1) model of the confirmed cases of COVID-19, and the remaining three data are used to test. From the definition 1, the first-order accumulating generated sequence is X^(1)^ = (291, 731, 1302, 2132, 3419, 5394, 8138, 12,653, 18,627, 26,338, 36,030, 47,821, 62,201, 79,406, 99,844, 124,168, 52,186).

*Step 2* System parameter estimation.

From theorem 2, and the values of X^(0)^ and X^(1)^, we calculate the matrix B and the matrix Y which are given by

$$B = \left( {\begin{array}{*{20}c} { - 511} & {2.3} & {1.5} & 1 \\ { - 1016.5} & {6.3} & {2.5} & 1 \\ { - 1717} & {12.3} & {3.5} & 1 \\ { - 2775.5} & {20.3} & {4.5} & 1 \\ { - 4406.5} & {30.3} & {5.5} & 1 \\ { - 6766} & {42.3} & {6.5} & 1 \\ { - 10395.5} & {56.3} & {7.5} & 1 \\ { - 15640} & {72.3} & {8.5} & 1 \\ { - 22482.5} & {90.3} & {9.5} & 1 \\ { - 31184} & {110.3} & {10.5} & 1 \\ { - 41925.5} & {132.3} & {11.5} & 1 \\ { - 55011} & {156.3} & {12.5} & 1 \\ { - 70803.5} & {182.3} & {13.5} & 1 \\ \end{array} } \right),\quad Y = \left( {\begin{array}{*{20}c} {440} \\ {571} \\ {830} \\ {1287} \\ {1975} \\ {2744} \\ {4515} \\ {5974} \\ {7711} \\ {9692} \\ {11791} \\ {14380} \\ {17205} \\ \end{array} } \right).$$

With the help of the Eq. (17), we can obtain the values of system parameters as

$$\left\{ {\begin{array}{*{20}c} {a = 0.0116,} \\ {b = 132.7801,} \\ {c = - 536.6728,} \\ {d = 1008.4680.} \\ \end{array} } \right.$$

*Step 3* Model construction.

Substituting the system parameters *a*, *b*, *c* and *d* into Eq. (12), we obtain that.

$$\frac{{dx^{\left( 1 \right)} \left( t \right)}}{dt} + 0.0116x^{\left( 1 \right)} \left( t \right) = 132.7801t^{2} - 536.6728t + 1008.4680$$.

And then we can obtain the expressions of Eq. (20) and Eq. (21), respectively. Therefore, we can compute the simulation and prediction values of the confirmed cases of COVID-19 of China. By a similar argument to the other grey forecasting models which are provided below.

The GM(1,1) model.

We can obtain system parameters *a* = -0.2441, *b* = 1116.9454 of the GM(1,1) model by the least squares method. And then the mathematical expression is given by.

$$\frac{{dx^{\left( 1 \right)} \left( t \right)}}{dt} - 0.2441x^{\left( 1 \right)} \left( t \right) = 1116.9454$$.

The DGM(1,1) model.

We directly deduce system parameters *a* = 0.2441, *b* = 1116.9454 of the DGM(1,1) model. And the mathematical formula is given by.

$$x^{\left( 1 \right)} \left( k \right) = 0.2441^{k - 1} x^{\left( 0 \right)} \left( 1 \right) + \frac{{1 - 0.2441^{k - 1} }}{0.7559} \times 1116.9454$$.

The NGM(1,1,*k*,*c*) model.

We can derive system parameters *a* = -0.1719, *b* = 463.7776 and *c* = -1124.6229 of the NGM(1,1,k,c) model. The whitening equation is built, there is.

$$\frac{{dx^{\left( 1 \right)} \left( t \right)}}{dt} - 0.1719x^{\left( 1 \right)} \left( t \right) = 463.7776t - 1124.6229$$.

The GVM(1,1) model.

We deduce system parameters *a* = -0.3820 and *b* = -2.0528E-6 of the GVM(1,1) model with the least squares estimation method. Further, the whitening equation is put forward, there is.

$$\frac{{dx^{\left( 1 \right)} \left( t \right)}}{dt} - 0.3820x^{\left( 1 \right)} \left( t \right) = - 0.000002\left( {x^{\left( 1 \right)} \left( t \right)} \right)^{2}$$.

The polynomial regression model.

We compute the values of parameters of the polynomial regression model where *a* = 120.9911, *b* = − 535.4727 and *c* = 916.0495, respectively. And then the mathematical expression is.

$$x^{\left( 0 \right)} \left( t \right) = 120.9911t^{2} - 535.4727t + 916.0495$$.

**Figure 4:**
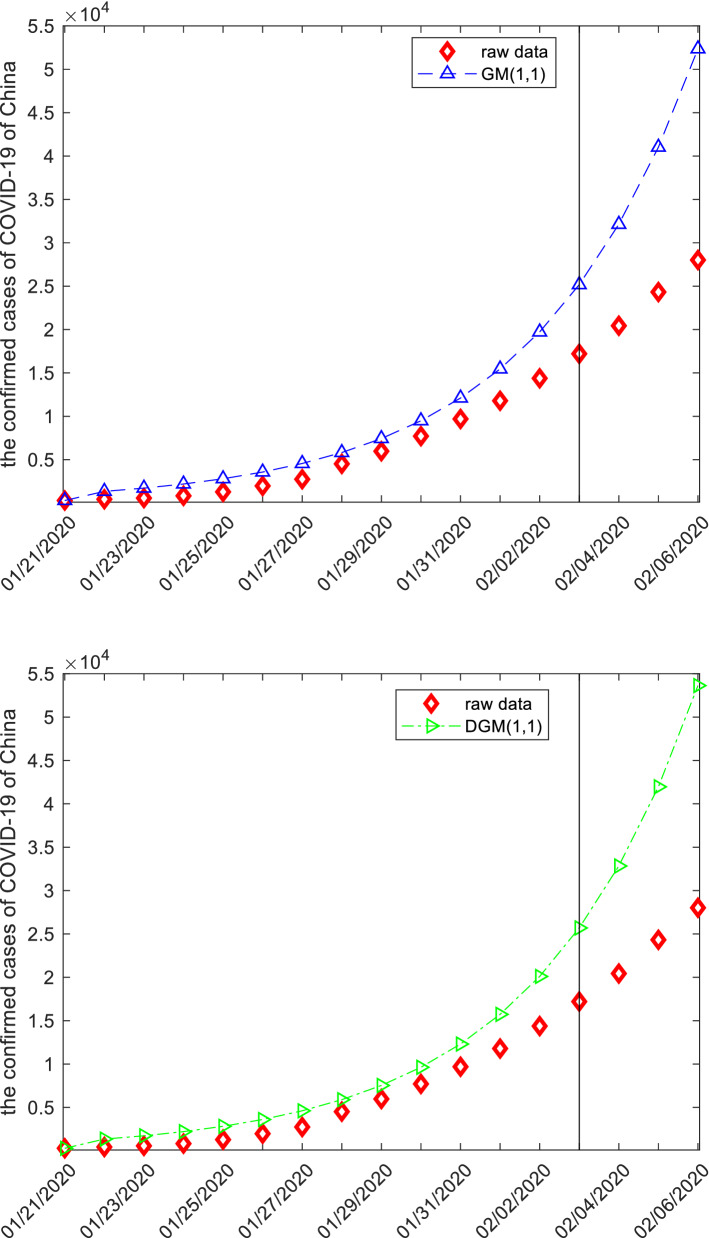

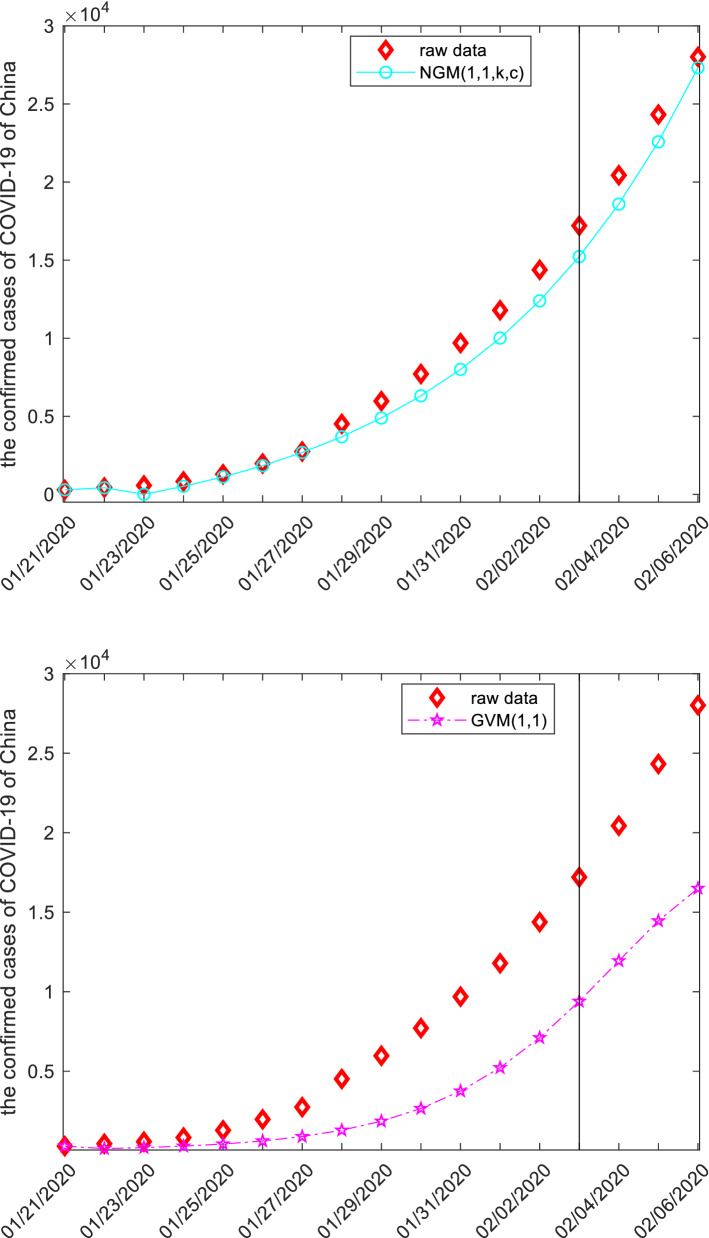

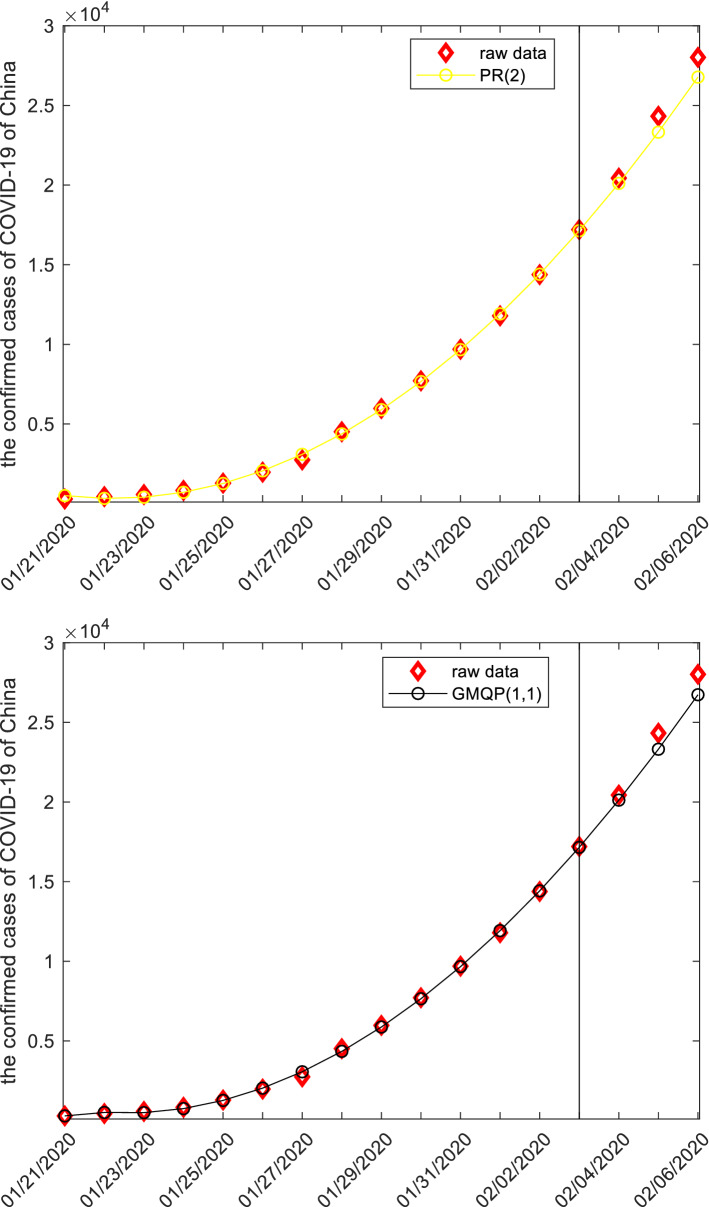
The plots of the confirmed cases of COVID-19 of China.

Once the specific grey forecasting models are established, the computational results and error metrics can be easily obtained which are displayed in the following Tables 9, 10, 11 and Fig. 4. The MAE_sim_, MAE_fit_, and MAE_all_ of the GMQP(1,1) model are 93.9043%, 871.5592% and 239.7146%, the MSE_sim_, MSE_fit_, and MSE_all_ of the GMQP(1,1) model are 14,610.4784%, 924,128.4138% and 185,145.0913%, the MAPE_sim_, MAPE_fit_, and MAPE_all_ of the GMQP(1,1) model are 4.8534%, 3.4346%, 4.5873%, the RMSE_sim_, RMSE_fit_, and RMSE_all_ of the GMQP(1,1) model are 7.1669%, 3.6842%, 6.6542%, the IA_sim_, IA_fit_, and IA_all_ of the GMQP(1,1) model are 0.9999%, 0.9990% and 0.9994%, the R_sim_, R_fit_, and R_all_ of the GMQP(1,1) model are 0.9998%, 0.9994% and 0.9996%, respectively.

**Table 9:** The computational results of the confirmed cases of COVID-19 of China.

date	data	GM(1,1)	DGM(1,1)	NGM(1,1,k,c)	GVM(1,1)	PR(2)	GMQP(1,1)
01/21/2020	291	291.0000	291.0000	291.0000	291.0000	501.5679	291.0000
01/22/2020	440	1345.5155	1353.1171	-420.6949	135.0533	329.0684	506.8141
01/23/2020	571	1717.4961	1729.3126	6.4145	197.5213	398.5511	495.6951
01/24/2020	830	2192.3143	2210.0987	513.6543	288.6436	710.0159	748.7279
01/25/2020	1287	2798.4006	2824.5535	1116.0578	421.2917	1263.4629	1262.8613
01/26/2020	1975	3572.0452	3609.8399	1831.4788	613.8116	2058.8920	2035.0789
01/27/2020	2744	4559.5712	4613.4528	2681.1206	892.0052	3096.3033	3062.3995
01/28/2020	4515	5820.1083	5896.0917	3690.1645	1291.4374	4375.6967	4341.8762
01/29/2020	5974	7429.1329	7535.3316	4888.5160	1859.6303	5897.0723	5870.5960
01/30/2020	7711	9482.9878	9630.3154	6311.6915	2657.0277	7660.4299	7645.6799
01/31/2020	9692	12,104.6504	12,307.7496	8001.8702	3754.3856	9665.7698	9664.2818
02/01/2020	11,791	15,451.0968	15,729.5679	10,009.1449	5222.4836	11,913.0918	11,923.5887
02/02/2020	14,380	19,722.7003	20,102.7252	12,393.0063	7108.5108	14,402.3959	14,420.8200
02/03/2020	17,205	25,175.2294	25,691.7139	15,224.1062	9394.7525	17,133.6821	17,153.2273
02/04/2020	20,438	32,135.1624	32,834.5614	18,586.3514	11,944.3084	20,106.9505	20,118.0938
02/05/2020	24,324	41,019.2354	41,963.2736	22,579.3906	14,459.3349	23,322.2011	23,312.7343
02/06/2020	28,018	52,359.3953	53,629.9636	27,321.5676	16,500.4091	26,779.4338	26,734.4943

**Table 10:** The APEs of different model in the confirmed cases of COVID-19 of China, (%).

date	GM(1,1)	DGM(1,1)	NGM(1,1,k,c)	GVM(1,1)	PR(2)	GMQP(1,1)
01/21/2020	0.0000	0.0000	0.0000	0.0000	72.3601	0.0000
01/22/2020	205.7990	207.5266	195.6125	69.3061	25.2117	15.1850
01/23/2020	200.7874	202.8569	98.8766	65.4078	30.2012	13.1883
01/24/2020	164.1343	166.2769	38.1139	65.2237	14.4559	9.7918
01/25/2020	117.4359	119.4680	13.2822	67.2656	1.8288	1.8756
01/26/2020	80.8630	82.7767	7.2669	68.9209	4.2477	3.0420
01/27/2020	66.1651	68.1287	2.2915	67.4925	12.8390	11.6035
01/28/2020	28.9061	30.5890	18.2688	71.3967	3.0853	3.8344
01/29/2020	24.3578	26.1354	18.1701	68.8713	1.2877	1.7309
01/30/2020	22.9800	24.8906	18.1469	65.5424	0.6558	0.8471
01/31/2020	24.8932	26.9887	17.4384	61.2630	0.2706	0.2860
02/01/2020	31.0414	33.4032	15.1120	55.7079	1.0355	1.1245
02/02/2020	37.1537	39.7964	13.8178	50.5667	0.1557	0.2839
02/03/2020	46.3251	49.3270	11.5135	45.3952	0.4145	0.3009
02/04/2020	57.2324	60.6545	9.0598	41.5583	1.6198	1.5653
02/05/2020	68.6369	72.5180	7.1724	40.5553	4.1186	4.1575
02/06/2020	86.8777	91.4125	2.4857	41.1078	4.4206	4.5810

**Table 11:** The evaluation measures of different forecasting models in the confirmed cases.

	GM(1,1)	DGM(1,1)	NGM(1,1,k,c)	GVM(1,1)	PR(2)	GMQP(1,1)
MAE_sim_	2481.2499	2624.5284	989.8823	3482.9573	105.5334	93.9043
MAE_fit_	17,577.9310	18,549.2662	1430.8968	9958.6492	857.1382	871.5592
MAE_all_	5311.8776	5610.4168	1072.5725	4697.1496	246.4593	239.7146
MSE_sim_	10,028,334.6309	11,366,851.6025	1,456,440.3220	19,361,149.2021	18,247.1500	14,610.4784
MSE_fit_	336,019,338.9233	373,597,128.8826	2,319,094.1103	100,703,105.1213	882,413.6768	924,128.4138
MSE_all_	71,151,647.9357	79,285,028.5926	1,618,187.9073	34,612,765.9370	180,278.3738	185,145.0913
MAPE_sim_	80.8340	82.9357	35.9932	63.2584	7.3607	4.8534
MAPE_fit_	70.9157	74.8617	6.2393	41.0738	3.3863	3.4346
MAPE_all_	78.9743	81.4218	30.4143	59.0988	6.6155	4.5873
RMSPE_sim_	104.2863	105.8779	62.9879	63.7160	12.2659	7.1669
RMSPE_fit_	71.9590	75.9256	6.8240	41.0759	3.6115	3.6842
RMSPEE_all_	99.0321	100.9411	56.8533	60.1239	11.1664	6.6542
IA_sim_	0.9414	0.9348	0.9903	0.8794	0.9999	0.9999
IA_fit_	0.8629	0.8536	0.9974	0.7769	0.9990	0.9990
IA_all_	0.8812	0.8721	0.9944	0.8391	0.9994	0.9994
R_sim_	0.9932	0.9930	0.9982	0.9811	0.9997	0.9998
R_fit_	0.9964	0.9964	0.9979	0.9990	0.9994	0.9994
R_all_	0.9858	0.9855	0.9979	0.9903	0.9995	0.9996

It follows from these results that the GM(1,1) model and the DGM(1,1) model has worst performance measures, the NGM(1,1,*k*,*c*) model and the GVM(1,1) model have worse performance measures, and the new model GMQP(1,1) have good performance measures. This also demonstrates that the grey model with quadratic polynomial term is more powerful to deal with the data of the confirmed cases of COVID-19 of China.

### The death cases from COVID-19 of China

**Figure 5:**
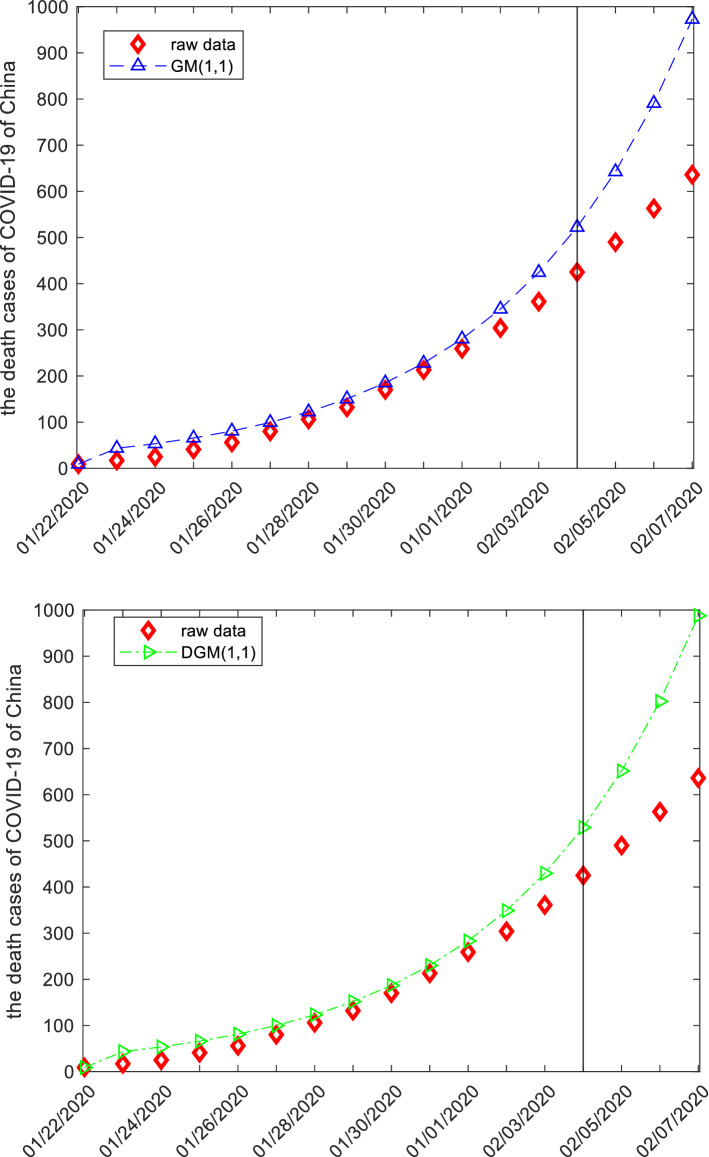

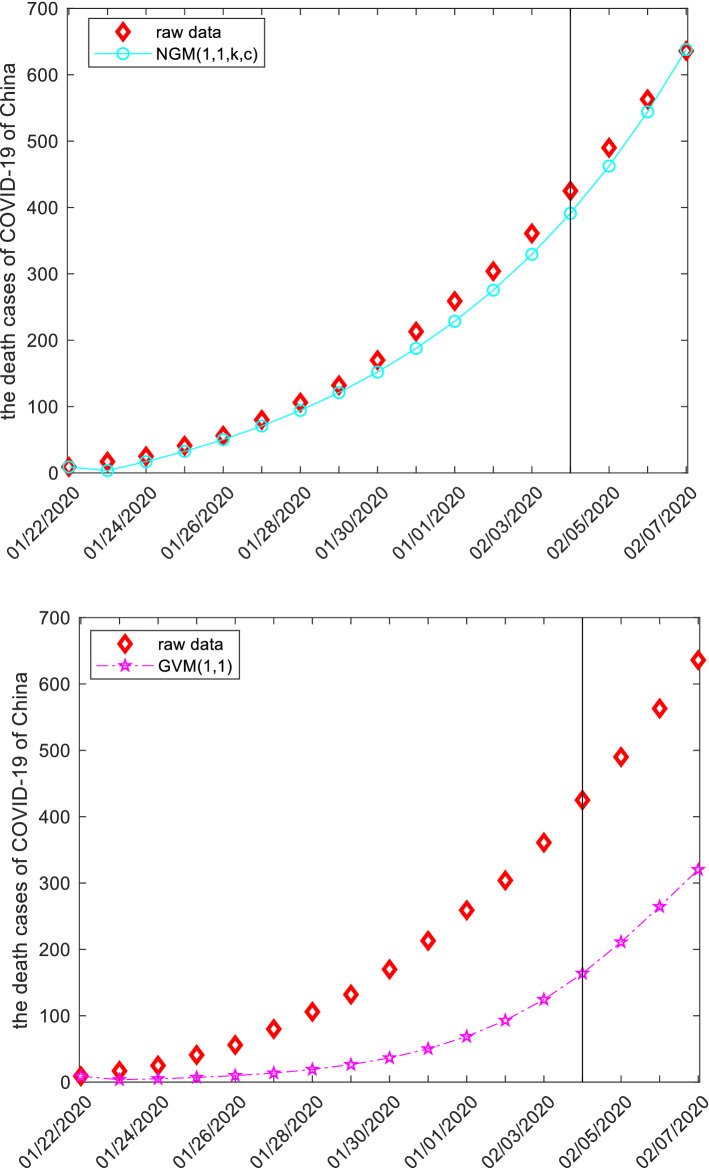

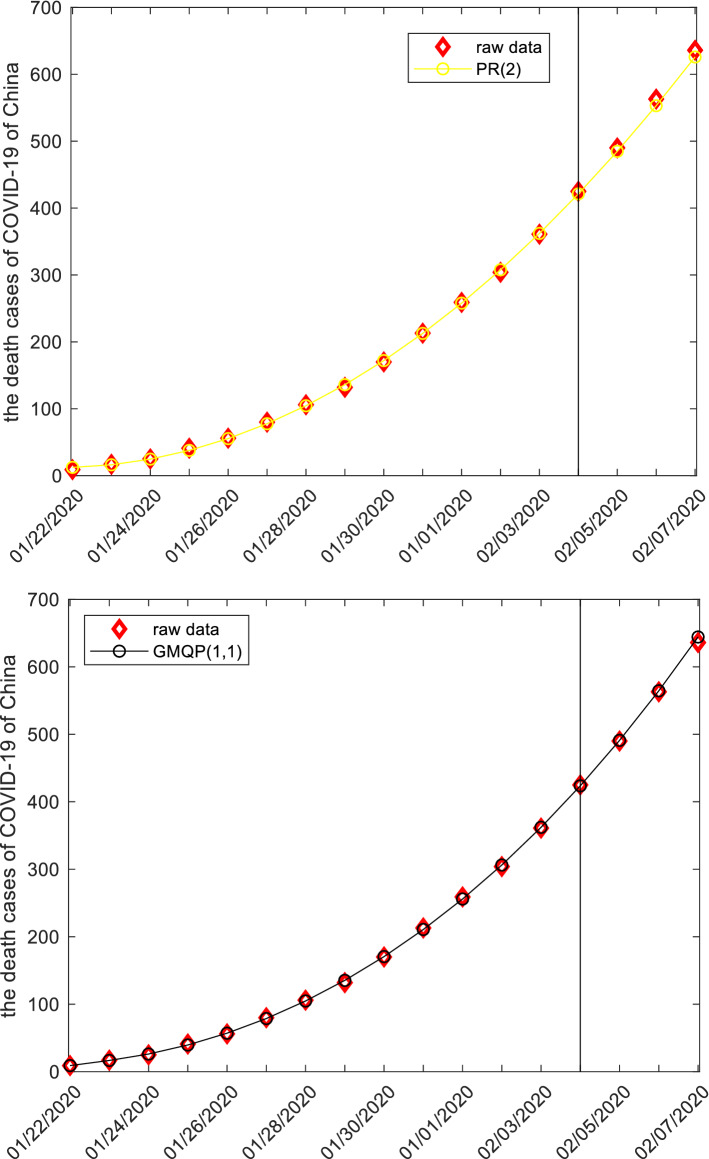
The plots of the death cases of COVID-19 of China.

This subsection discusses the death cases from COVID-19 of China by employing grey models. The raw data are collected from the website: http://www.nhc.gov.cn, and displayed in the following Tables 12, 13, 14 and Fig. 5. The first 14 observations are used to build models, and the left three observation is used to test. Similar argument is applied to derive system parameters of each model, and then the mathematical expressions are given below.

**Table 12:** The computational results of the death cases of COVID-19 of China.

date	data	GM(1,1)	DGM(1,1)	NGM(1,1,k,c)	GVM(1,1)	PR(2)	GMQP(1,1)
01/22/2020	9	9.0000	9.0000	9.0000	9.0000	12.3393	9.0000
01/23/2020	17	43.3505	43.5237	3.5662	3.5938	16.2047	16.6241
01/24/2020	25	53.3413	53.5946	17.0303	5.0240	24.6676	26.1427
01/25/2020	41	65.6348	65.9958	32.4983	7.0185	37.7280	39.5647
01/26/2020	56	80.7615	81.2664	50.2685	9.7958	55.3860	57.0369
01/27/2020	80	99.3744	100.0706	70.6835	13.6544	77.6415	78.7117
01/28/2020	106	122.2770	123.2258	94.1369	18.9982	104.4945	104.7470
01/29/2020	132	150.4579	151.7389	121.0809	26.3669	135.9451	135.3069
01/30/2020	170	185.1336	186.8495	152.0351	36.4658	171.9931	170.5615
01/31/2020	213	227.8010	230.0844	187.5962	50.1892	212.6387	210.6874
02/01/2020	259	280.3017	283.3233	228.4500	68.6193	257.8819	255.8677
02/02/2020	304	344.9022	348.8812	275.3842	92.9689	307.7225	306.2925
02/03/2020	361	424.3911	429.6083	329.3037	124.4206	362.1607	362.1591
02/04/2020	425	522.1995	529.0149	391.2482	163.8024	421.1964	423.6721
02/05/2020	490	642.5497	651.4229	462.4120	211.0520	484.8297	491.0438
02/06/2020	563	790.6366	802.1549	544.1674	264.5072	553.0604	564.4946
02/07/2020	636	972.8529	987.7645	638.0906	320.2373	625.8887	644.2531

**Table 13:** The Errors of different model in the death cases of COVID-19 of China, (%).

date	GM(1,1)	DGM(1,1)	NGM(1,1,k,c)	GVM(1,1)	PR(2)	GMQP(1,1)
01/22/2020	0.0000	0.0000	0.0000	0.0000	37.1032	0.0000
01/23/2020	155.0027	156.0217	79.0226	78.8599	4.6784	2.2112
01/24/2020	113.3654	114.3784	31.8788	79.9042	1.3297	4.5707
01/25/2020	60.0849	60.9653	20.7357	82.8816	7.9804	3.5008
01/26/2020	44.2170	45.1187	10.2348	82.5075	1.0964	1.8516
01/27/2020	24.2180	25.0882	11.6456	82.9321	2.9481	1.6104
01/28/2020	15.3557	16.2508	11.1916	82.0771	1.4203	1.1821
01/29/2020	13.9833	14.9537	8.2720	80.0251	2.9887	2.5052
01/30/2020	8.9021	9.9115	10.5676	78.5496	1.1724	0.3303
01/31/2020	6.9488	8.0208	11.9267	76.4370	0.1696	1.0857
02/01/2020	8.2246	9.3912	11.7954	73.5060	0.4317	1.2094
02/02/2020	13.4547	14.7635	9.4131	69.4181	1.2245	0.7541
02/03/2020	17.5599	19.0051	8.7801	65.5344	0.3215	0.3211
02/04/2020	22.8705	24.4741	7.9416	61.4583	0.8950	0.3124
02/05/2020	31.1326	32.9435	5.6302	56.9282	1.0552	0.2130
02/06/2020	40.4328	42.4787	3.3451	53.0183	1.7655	0.2655
02/07/2020	52.9643	55.3089	0.3287	49.6482	1.5898	1.2977

**Table 14:** The evaluation measures of different forecasting models in the death cases.

	GM(1,1)	DGM(1,1)	NGM(1,1,k,c)	GVM(1,1)	PR(2)	GMQP(1,1)
MAE_sim_	31.6097	33.7059	18.1322	120.6217	1.9217	1.5865
MAE_fit_	239.0131	250.7807	16.1704	297.7345	8.4071	3.5972
MAE_all_	70.4979	74.4075	17.7643	153.8304	3.1377	1.9635
MSE_sim_	1518.4319	1737.0336	427.6899	21,254.5680	5.4033	3.2758
MSE_fit_	62,853.2325	68,996.8802	373.3784	88,872.0233	75.9216	23.8122
MSE_all_	13,018.7071	14,348.2548	417.5065	33,932.8408	18.6255	7.1264
MAPE_sim_	38.7836	39.8725	17.9543	76.4685	2.0505	1.6496
MAPE_fit_	41.5099	43.5770	3.1013	53.1982	1.4702	0.5921
MAPE_all_	39.2948	40.5671	15.1693	72.1053	1.9417	1.4513
RMSPE_sim_	58.6176	59.3060	25.9388	76.7641	2.9375	2.0616
RMSPE_fit_	42.4628	44.5301	3.7858	53.2813	1.5009	0.7745
RMSPEE_all_	55.9451	56.8289	23.4383	72.9392	2.7265	1.8883
IA_sim_	0.9819	0.9794	0.9947	0.7925	0.9999	1.0000
IA_fit_	0.9271	0.9220	0.9992	0.4252	0.9998	0.9999
IA_all_	0.9433	0.9388	0.9972	0.6976	0.9999	1.0000
R_sim_	0.9948	0.9947	0.9996	0.9729	0.9998	0.9999
R_fit_	0.9982	0.9982	0.9992	0.9999	0.9998	0.9997
R_all_	0.9870	0.9868	0.9986	0.9756	0.9999	0.9999

The GM(1,1) model.

$$\frac{{dx^{\left( 1 \right)} \left( t \right)}}{dt} - 0.2074x^{\left( 1 \right)} \left( t \right) = 37.1439$$

The DGM(1,1) model.

$$x^{\left( 1 \right)} \left( k \right) = 0.2074^{k - 1} x^{\left( 0 \right)} \left( 1 \right) + \frac{{1 - 0.2074^{k - 1} }}{0.7926} \times 37.1439$$

The NGM(1,1,k,c) model.

$$\frac{{dx^{\left( 1 \right)} \left( t \right)}}{dt} - 0.1387x^{\left( 1 \right)} \left( t \right) = 12.0569t - 15.8702$$

The GVM(1,1) model.

$$\frac{{dx^{\left( 1 \right)} \left( t \right)}}{dt} - 0.3367x^{\left( 1 \right)} \left( t \right) = - 0.000065\left( {x^{\left( 1 \right)} \left( t \right)} \right)^{2}$$

The polynomial regression model.

$$x^{\left( 0 \right)} \left( t \right) = 2.298811t^{2} - 3.0309t + 13.0714$$

The GMQP(1,1) model.

$$\frac{{dx^{\left( 1 \right)} \left( t \right)}}{dt} - 0.0369x^{\left( 1 \right)} \left( t \right) = 9.2777t^{2} + 1.7801t + 1.7402$$

When the specific mathematical expression of each model is derived, the computational results and error metrics are straightforward obtained, which are provided in the following Tables 12, 13, 14 and Fig. 5. The MAE_sim_, MAE_fit_, and MAE_all_ of the GMQP(1,1) model are 1.5865%, 3.5972% and 1.9635%, the MSE_sim_, MSE_fit_, and MSE_all_ of the GMQP(1,1) model are 3.2758%, 23.8122% and 7.1264%, the MAPE_sim_, MAPE_fit_, and MAPE_all_ of the GMQP(1,1) model are 1.6496%, 0.5921%, 1.4513%, the RMSE_sim_, RMSE_fit_, and RMSE_all_ of the GMQP(1,1) model are 2.0616%, 0.7745%, 1.8883%, the IA_sim_, IA_fit_, and IA_all_ of the GMQP(1,1) model are 1.0000%, 0.9999% and 1.0000%, the R_sim_, R_fit_, and R_all_ of the GMQP(1,1) model are 0.9999%, 0.9997% and 0.9999%, respectively.

Similarly, the GM(1,1) model, the DGM(1,1) model and the GVM(1,1) model have the worst computational results, the NGM(1,1,*k*,*c*) model has the worse computational results, and the GMQP(1,1) has the most computational results. It indicates that the new model has higher precision than the other forecasting models in the death cases from COVID-19 of China.

### The recovered cases from COVID-19 in China

**Figure 6:**
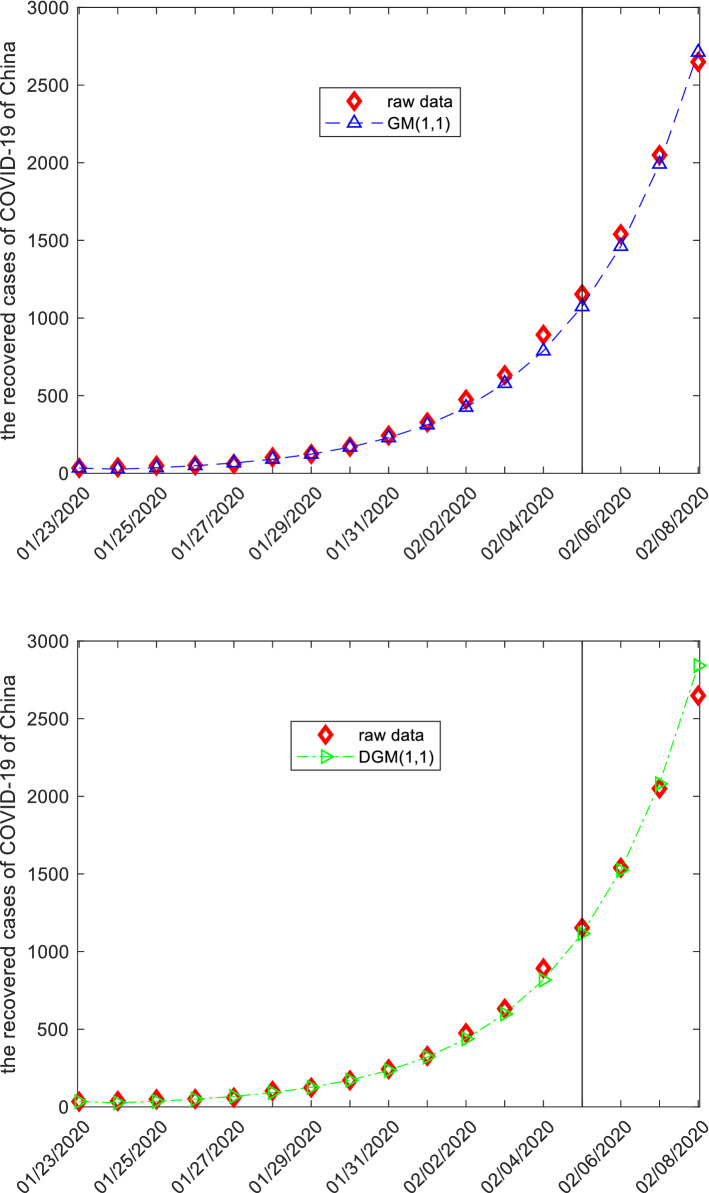

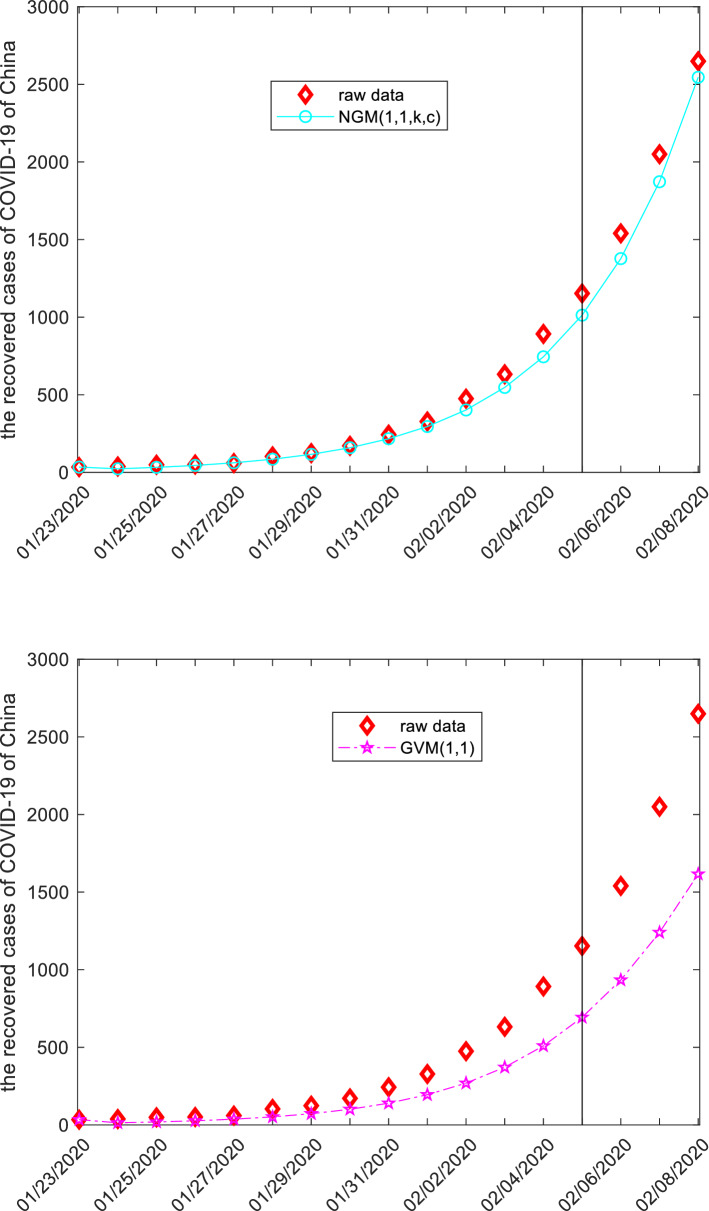

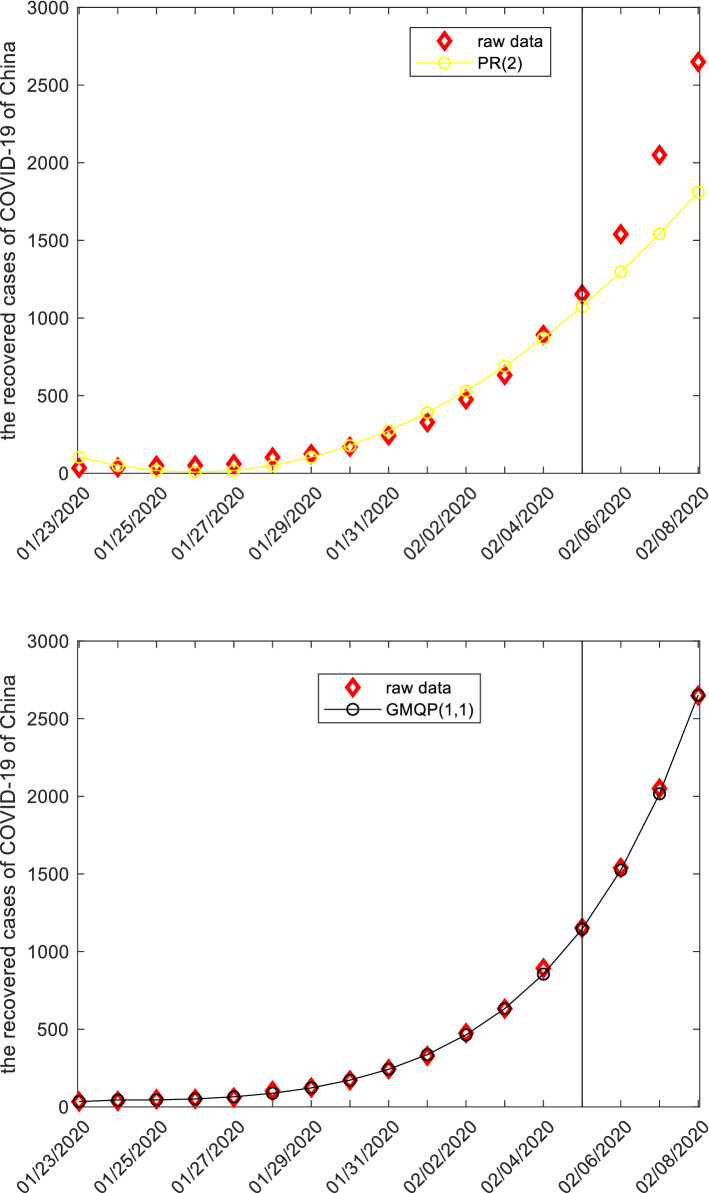
The plots of the recovered cases of COVID-19 of China.

This subsection discusses the recovered cases from COVID-19 of China by employing grey models. The raw data are collected from the website: http://www.nhc.gov.cn, and displayed in the following Tables 15, 16, 17 and Fig. 6. The first 14 observations are used to build models, and the left three observation is used to test. Similar argument is applied to derive system parameters of each model, and then the mathematical expressions are given below.

**Table 15:** The computational results of the recovered cases of COVID-19 of China.

date	data	GM(1,1)	DGM(1,1)	NGM(1,1,k,c)	GVM(1,1)	PR(2)	GMQP(1,1)
01/23/2020	34	34.0000	34.0000	34.0000	34.0000	103.0464	34.0000
01/24/2020	38	26.2967	26.5439	23.0438	13.5607	49.7041	44.3017
01/25/2020	49	35.8201	36.2471	32.2317	18.9614	17.6929	44.9811
01/26/2020	51	48.7923	49.4974	44.7177	26.5053	7.0126	51.2829
01/27/2020	60	66.4624	67.5913	61.6854	37.0357	17.6635	64.8046
01/28/2020	103	90.5316	92.2996	84.7437	51.7208	49.6453	87.5974
01/29/2020	124	123.3176	126.0400	116.0788	72.1723	102.9582	122.2954
01/30/2020	171	167.9769	172.1143	158.6615	100.6009	177.6022	172.2811
01/31/2020	243	228.8096	235.0313	216.5292	140.0143	273.5772	241.8978
02/01/2020	328	311.6728	320.9477	295.1684	194.4569	390.8832	336.7231
02/02/2020	475	424.5448	438.2711	402.0350	269.2765	529.5203	463.9190
02/03/2020	632	578.2933	598.4826	547.2611	371.3702	689.4885	632.6827
02/04/2020	892	787.7216	817.2598	744.6160	509.3095	870.7876	854.8239
02/05/2020	1153	1072.9942	1116.0117	1012.8110	693.1414	1073.4179	1145.5082
02/06/2020	1540	1461.5780	1523.9734	1377.2742	933.5335	1297.3791	1524.2097
02/07/2020	2050	1990.8871	2081.0667	1872.5608	1239.7818	1542.6714	2015.9351
02/08/2021	2649	2711.8848	2841.8073	2545.6297	1616.1719	1809.2948	2652.7965

**Table 16:** The Errors of different model in the recovered cases of COVID-19 of China, (%).

date	GM(1,1)	DGM(1,1)	NGM(1,1,k,c)	GVM(1,1)	PR(2)	GMQP(1,1)
01/23/2020	0.0000	0.0000	0.0000	0.0000	203.0777	0.0000
01/24/2020	30.7980	30.1476	39.3585	64.3139	30.8003	16.5833
01/25/2020	26.8978	26.0262	34.2210	61.3033	63.8921	8.2019
01/26/2020	4.3288	2.9463	12.3183	48.0289	86.2497	0.5547
01/27/2020	10.7706	12.6522	2.8091	38.2738	70.5609	8.0076
01/28/2020	12.1052	10.3888	17.7245	49.7856	51.8007	14.9540
01/29/2020	0.5503	1.6452	6.3881	41.7965	16.9692	1.3747
01/30/2020	1.7679	0.6516	7.2155	41.1691	3.8609	0.7492
01/31/2020	5.8397	3.2793	10.8933	42.3810	12.5832	0.4536
02/01/2020	4.9778	2.1501	10.0096	40.7143	19.1717	2.6595
02/02/2020	10.6221	7.7324	15.3611	43.3102	11.4780	2.3328
02/03/2020	8.4979	5.3034	13.4080	41.2389	9.0963	0.1080
02/04/2020	11.6904	8.3789	16.5229	42.9025	2.3781	4.1677
02/05/2020	6.9389	3.2080	12.1586	39.8837	6.9022	0.6498
02/06/2020	5.0923	1.0407	10.5666	39.3809	15.7546	1.0253
02/07/2020	2.8836	1.5154	8.6556	39.5228	24.7477	1.6617
02/08/2020	2.3739	7.2785	3.9022	38.9894	31.6990	0.1433

**Table 17:** The evaluation measures of different forecasting models in the recovered cases.

	GM(1,1)	DGM(1,1)	NGM(1,1,k,c)	GVM(1,1)	PR(2)	GMQP(1,1)
MAE_sim_	28.3608	18.7810	44.8298	140.0672	39.7383	7.6964
MAE_fit_	66.8066	79.9669	147.8451	816.5043	529.8849	17.8839
MAE_all_	35.5694	30.2534	64.1452	266.8992	131.6408	9.6065
MSE_sim_	1822.7206	770.2744	4365.6633	39,193.1139	2009.7999	150.7354
MSE_fit_	4532.9501	12,798.8826	22,883.2485	696,996.3883	340,450.6755	474.7209
MSE_all_	2330.8886	3025.6384	7837.7105	162,531.2278	65,467.4640	211.4826
MAPE_sim_	10.4450	8.8085	15.2606	45.7771	29.6726	4.6767
MAPE_fit_	3.4499	3.2782	7.7081	39.2977	24.0671	0.9435
MAPE_all_	9.1335	7.7715	13.8446	44.5622	28.6215	3.9767
RMSPE_sim_	13.5461	12.5571	18.2762	46.4493	40.4100	7.1431
RMSPE_fit_	3.6461	4.3342	8.2016	39.2984	24.9365	1.1304
RMSPEE_all_	12.3119	11.4733	16.8524	45.1948	37.9918	6.4573
IA_sim_	0.9977	0.9990	0.9946	0.9530	0.9975	0.9998
IA_fit_	0.9995	0.9987	0.9973	0.8587	0.9444	0.9999
IA_all_	0.9990	0.9988	0.9965	0.8986	0.9639	0.9999
R_sim_	0.9993	0.9993	0.9993	0.9992	0.9905	0.9996
R_fit_	0.9991	0.9991	0.9991	0.9999	0.9998	0.9996
R_all_	0.9987	0.9985	0.9988	0.9998	0.9832	0.9999

The GM(1,1) model.

$$\frac{{dx^{\left( 1 \right)} \left( t \right)}}{dt} - 0.3091x^{\left( 1 \right)} \left( t \right) = 11.9339$$

The DGM(1,1) model.

$$x^{\left( 1 \right)} \left( k \right) = 0.3091^{k - 1} x^{\left( 0 \right)} \left( 1 \right) + \frac{{1 - 0.3091^{k - 1} }}{0.6909} \times 11.9339$$

The NGM(1,1,*k*,*c*) model.

$$\frac{{dx^{\left( 1 \right)} \left( t \right)}}{dt} - 0.3067x^{\left( 1 \right)} \left( t \right) = 0.7831t + 8.1075$$

The GVM(1,1) model.

$$\frac{{dx^{\left( 1 \right)} \left( t \right)}}{dt} - 0.3359x^{\left( 1 \right)} \left( t \right) = - 0.000007\left( {x^{\left( 1 \right)} \left( t \right)} \right)^{2}$$

The polynomial regression model.

$$x^{\left( 0 \right)} \left( t \right) = 10.6655t^{2} - 85.3389t + 177.7198$$

The GMQP(1,1) model.

$$\frac{{dx^{\left( 1 \right)} \left( t \right)}}{dt} - 0.2501x^{\left( 1 \right)} \left( t \right) = 54.5571t^{2} - 19.9387t + 2.3094$$

When the specific mathematical expression of each model is derived, the computational results and error metrics are straightforward obtained, which are provided in the following Tables 15, 16, 17 and Fig. 6, respectively. The MAE_sim_, MAE_fit_, and MAE_all_ of the GMQP(1,1) model are 7.6964%, 17.8839% and 9.6065%, the MSE_sim_, MSE_fit_, and MSE_all_ of the GMQP(1,1) model are 150.7354%, 474.7209% and 211.4826%, the MAPE_sim_, MAPE_fit_, and MAPE_all_ of the GMQP(1,1) model are 4.6767%, 0.9435%, 3.9767%, the RMSE_sim_, RMSE_fit_, and RMSE_all_ of the GMQP(1,1) model are 7.1431%, 1.1304%, 6.4573%, the IA_sim_, IA_fit_, and IA_all_ of the GMQP(1,1) model are 0.9998%, 0.9999% and 0.9999%, the R_sim_, R_fit_, and R_all_ of the GMQP(1,1) model are 0.9996%, 0.9996% and 0.9999%, respectively.

Similarly, the GVM(1,1) model has the worst computational results, the GM(1,1) model, the DGM(1,1) model and the NGM(1,1,*k*,*c*) model have the better computational results, and the GMQP(1,1) has the most best computational results. It indicates that the new model has higher precision than the other forecasting models in the recovered cases from COVID-19 of China.

## Conclusion

This paper studied the grey forecasting model with quadratic polynomial term, and applied it to the confirmed cases, the death cases and the recovered cases from COVID-19 of China at the early stage. By using the grey technique and some mathematical derivations, the grey basic form, the time response function and the restored values are all systematically analyzed. With raw datasets of COVID-19 in China, we compute the simulation and fitting values by different forecasting models. It follows from the computational results, we can observed the new model has higher precision than other models. This also implied that our generalized model has applicable value in the COVID-19.

In this work, the GMQP(1,1) model is an univariate grey forecasting model and some factors such as social isolation and lockdown, vaccines, active treatment cannot be considered. In addition, the integer order accumulating generated operation is used to preprocess the raw data. It is generally known that the fractional order accumulating generated operation or the new information priority to preprocess raw data can get more accurate results. Thus in the future, we will continuous consider such a model with other accumulating generated operator including new information priority, fractional accumulating generated operator. Further, other multivariate grey forecasting models can be constructed to study the COVID-19.

## Data Availability

The data used to support the findings of this study are available from the corresponding author upon request.
